# Transcriptomic and Epigenetic Profiling of the Lung of Influenza-Infected Pigs: A Comparison of Different Birth Weight and Susceptibility Groups

**DOI:** 10.1371/journal.pone.0138653

**Published:** 2015-09-22

**Authors:** Jamie M. Wilkinson, Rayna E. Gunvaldsen, Susan E. Detmer, Michael K. Dyck, Walter T. Dixon, George R. Foxcroft, Graham S. Plastow, John C. S. Harding

**Affiliations:** 1 Department of Agricultural, Food, and Nutritional Science, University of Alberta, Edmonton, Alberta, Canada; 2 Department of Large Animal Clinical Sciences, Western College of Veterinary Medicine, University of Saskatchewan, Saskatoon, SK, Canada; University of Hong Kong, HONG KONG

## Abstract

Influenza viruses are a common cause of respiratory disease in swine. Infections range in severity from asymptomatic to causing significant morbidity. The main objective of this study was to compare lung transcriptomic and epigenetic responses to influenza infection in pigs from high or low birth weight litters. The latter is a potential indicator of intrauterine growth restriction, a significant risk factor for prenatal programming effects. Individual pigs from high (HBW) or low birth weight (LBW) litters (n = 17) were inoculated with influenza A virus and euthanized 48 hours later. Lesion severity and viral loads were assessed as previously described. The transcriptional response to infection in LBW and HBW groups (n = 16) was assessed by microarray. A separate analysis of pigs classified as ‘Resilient’ (RES) or ‘Susceptible’ (SUS) (n = 6) on the basis of severity of lung pathology was also conducted. Eight genes were confirmed as differentially expressed for the birth weight comparison, including three antiviral genes with lower expression in LBW: *ISG15*, *OAS1*, and *OAS2* (*P*<0.05). The promoter region methylation status of these three genes was assessed for each birth weight group, and no differences were found. These expression data are consistent with our previous finding that LBW pigs had less severe lesion scores and a trend towards lower viral titres in lung than the HBW cohort. The SUS v RES comparison identified 91 differentially expressed genes (FDR<0.05) that were enriched with functional annotation terms and pathways associated with inflammation. The cytokine genes *IL6*, *IL8*, and *CCL2* were all upregulated in SUS pigs, and may have driven disease severity in these animals. In conclusion, this study found no evidence that the transcriptional immune response to influenza was adversely affected by low litter birth weight, but did identify several candidate genes for driving disease pathology.

## Introduction

Resilience in the face of infectious disease is an increasingly desirable trait in today’s intensive pig farming industry. However, it is rare to find naïve individuals that exhibit complete resistance to a particular infectious disease within a population. For most diseases, the reality is a range of variable susceptibility to infection, disease pathology, or both that is governed by multiple genetic and environmental factors. Influenza is a highly contagious disease that can rapidly infect all naïve pigs on a farm. However, the severity of clinical signs and pathology can vary significantly among individuals within a population. The infection can be subclinical and asymptomatic in some individuals, but cause acute respiratory distress and morbidity, and occasionally death, in others [1].

Multiple host factors have an impact on susceptibility to infectious disease. One factor that has not previously been studied in swine is that of ‘prenatal programming’ of postnatal disease susceptibility. Specific periods of prenatal development are critically important in determining the postnatal functional attributes of the tissues and hence biological systems of an individual. Fetal development is constrained by the *in utero* environment, and the developing fetus can adaptively adjust its development in response to the environmental cues it receives. This ‘programming’ of biological systems can have adverse consequences after birth, as adaptive changes made to these systems during development have the potential to persist into adulthood, and even across generations [2].

An extensive body of literature has been published on the prenatal programming of chronic conditions in adulthood such as diabetes and cardiovascular disease [3], but relatively little attention has been paid to the potential for programming effects on the developing immune system. A limited number of epidemiological studies on individuals from developing countries who were born small for gestational age (SGA), an indirect measure of nutrient restriction *in utero*, have found a reduction in cellular and adaptive immune responses to vaccination [4,5], diminished thymic function [6], and increased risk of death by infectious diseases [7]. In addition, experiments in rodent and farm animal species have generally found that stressors applied to the mother during gestation are associated with a reduction in the immune function of the offspring [8,9].

In the pig, intrauterine crowding (IUC) during gestation is a major risk factor for prenatal programming. Experimental models have shown that the degree of IUC during the early period of gestation restricts placental development, with subsequent knock-on effects on embryonic and fetal development [10–12]. In contemporary swine populations, a proportion of sows repeatedly produce low birth weight litters across multiple parities, probably due to IUC caused by a combination of high ovulation rates, high early embryonic survival, and limited uterine capacity [13]. The piglets from these litters exhibit some of the morphometric deviations from normal allometric growth that are characteristic of intrauterine growth restriction (IUGR), and grow more slowly throughout the production phase than piglets from normal weight litters [14]. However, direct evidence for the effect of low litter birth weight on immune parameters and disease severity following infection in pigs is lacking.

The main objective of this study was to use transcriptomic and epigenetic approaches to compare the responses in lung tissue following an influenza A virus challenge to piglets originating from either ‘high’ or ‘low’ birth weight litters. Phenotypic differences in the response to infection between these high and low birth weight pigs have previously been described [15]. The epigenetic modification of gene promoters, with its subsequent effect on transcription, is thought to be the molecular basis for initiating and maintaining programming effects, but very few studies have actually investigated programming effects at the molecular level. A second objective was to compare pigs classified as ‘resilient’ or ‘susceptible’ to disease to identify candidate genes and pathways involved in influenza pathology. Pigs were selected on the basis of severity of lung pathology.

## Materials and Methods

### Animal Selection and Housing

A detailed description of the animal experiment from which lung tissue was obtained for the present study has been published previously [15]. Briefly, 17 high birth weight (HBW) and 17 low birth weight (LBW) litters from first and second parity dams were identified at birth based on the average weight of all live and stillborn piglets in the litter being ≥ 0.7 SD or ≤ -0.7 SD, respectively (Z-scores), from the farm’s historical mean litter birth weight for a given litter size and parity. Two male piglets with birth weights close to the mean for their litter of origin were selected at weaning (3 weeks of age) from each HBW and LBW litter and transported from the farm to a biocontainment level 2 facility at the University of Saskatchewan. Prior to transportation, all piglets were confirmed negative for serum antibodies to swine influenza A virus. After arrival at the facility, a nasal swab was collected and confirmed negative for influenza A by reverse transcription polymerase chain reaction (RT-PCR) [16]. Pigs were acclimated at the facility for 5 days prior to influenza challenge.

### Influenza Challenge, Sampling, and Clinical and Pathological Assessments

Pigs were inoculated intratracheally with 2 ml of influenza strain A/swine/Texas/4199-2/98 H3N2 (TX98) at a titre of 1x10^6.3^ TCID_50_/ml. Rectal temperatures and clinical signs of respiratory disease were recorded twice daily during the challenge period. Pigs were euthanized 48 hours post-inoculation by intravenous pentobarbital injection. A humane intervention point protocol (HIP) was in place for this study requiring that animals with any of the following clinical signs were to be euthanized immediately: body temperatures >41°C or <35°C for a 24 h period, in lateral recumbency and non-responsive, severe dyspnea, severe depression, severe dehydration, painful or life-threatening fracture or injury. No pigs, however, met these criteria. Lungs and trachea were removed and the percentage of lung with lesions typical of influenza A (purple, lobular consolidation) was evaluated. These data were used to evaluate apparent differences among birth weight phenotypes in disease severity and also to identify two groups (n = 6 each) with divergent severity of influenza pneumonia, classified based on percentage lung area affected by consolidation, as ‘Resilient’ (RES; 0 ±0%) and ‘Susceptible’ (SUS; 27.67 ±11.57%). Tissue samples from each cranial and middle lobe were snap-frozen in liquid nitrogen within 15 minutes of death and stored at -80°C for gene expression studies. Similar samples were fixed in 10% (v/v) formalin and processed routinely for haematoxylin and eosin staining to score microscopic lesion severity and immunohistochemistry with anti-influenza A ribonucleoprotein antibody (National Institute of Allergy and Infectious Disease, Bethesda, ND, USA) to quantify immunoreactivity to influenza antigen [17]. This work was approved by the University of Saskatchewan’s Animal Research Ethics Board, and adhered to the Canadian Council on Animal Care guidelines for humane animal use (protocol 20090157).

### Nucleic Acid Extractions

Total RNA and genomic DNA were extracted from cranial lung tissue using the All Prep DNA/RNA mini kit (Qiagen; Hilden, Germany) according to the manufacturer’s instructions. Nucleic acids were quantified and assessed for purity by spectrophotometry using a Nanodrop ND 2000 (Thermo Fisher Scientific; Waltham, USA). All samples had 260/280 nm absorbance ratios of ~2. RNA quality was assessed using an Agilent Bioanalyzer (Agilent Technologies; Santa Clara, USA). The mean RNA Integrity Number (RIN) sample value was 8.2 ±0.09.

### Microarray Hybridization

One pig from each of 32 litters (16 HBW and 16 LBW) was randomly selected for microarray analysis. RNA samples from 2 litters were excluded on the basis of a RIN <7. Lung RNA of individual animals, and a common reference that included RNA from 3 swine tissues pooled from experiment animals (lung, lymph node, and thymus), were hybridized to each array (32 hybridizations in total). A 1μg amount of total RNA was reverse transcribed into cDNA, which was then used to synthesize antisense RNA (aRNA) by *in vitro* transcription. Two micrograms of aRNA were then labeled in an ozone-free environment with either Cy3 or Cy5 using the Universal Linkage System (ULS^TM^). Reverse transcription, aRNA synthesis, and labeling were all performed using the RNAampULSe kit according to the manufacturer’s instructions (Kreatech Diagnostics; Amsterdam, The Netherlands). Labeled aRNAs were purified using the PicoPure RNA Isolation kit (Life Technologies; Carlsbad, USA) and 825 ng of each dye-labeled aRNA was hybridized for 17 h to Porcine V2 Gene Expression 4x44 Microarrays (Agilent Technologies). Microarray slides were washed (Gene Expression Wash Pack; Agilent Technologies) and then scanned on an Axon AL4200 scanner (Molecular Devices; Sunnyvale, USA). Hybridization signal intensities were quantified using GenePixPro v7.0 software (Molecular Devices). No flagging criteria were applied to the spots.

### Transcriptomic Analyses

Statistical analysis of the microarray data to identify differentially expressed genes (DEGs) was carried out using the ‘limma’ package (Smyth; 2005) in the program ‘FlexArray’ (http://www.gqinnovationcenter.com/services/bioinformatics/flexarray/index.aspx?l=e). Firstly, background subtraction was performed on the raw expression data from each array using a ‘norm exp’ method. Next, the background-subtracted expression data were normalized both within array (using a global loess method) and between arrays (using a scale normalization method). The ‘limma simple’ algorithm was then used to assess differential gene expression between the HBW and LBW groups, and also between the RES and SUS groups. *P* values were corrected for multiple-testing error using the False Discovery Rate (FDR) method. Only genes with a ≥1.75 fold expression difference, and an appropriate *P* value, were considered as differentially expressed. For the LBW v HBW comparison, no genes were found to be differentially expressed after application of the FDR correction, so an uncorrected *P* value cutoff of ≤0.05 was adopted. For the SUS v RES comparison, a ≥1.75 fold expression difference and an FDR cutoff of ≤0.05 were used. All data have been submitted to the NCBI Geo database (series identifiers GSE62765 for the LBW v HBW contrast and GSE62768 for the SUS v RES contrast).

### Gene Set Enrichment Analysis (GSEA)

Normalized expression ratio data for the LBW v HBW and SUS v RES contrasts were further analyzed using the Gene Set Enrichment Analysis (GSEA) method [18]. This approach evaluates microarray data at the level of gene sets rather than individual genes. This helps to reduce the limitations imposed by the stringent multiple hypothesis testing corrections applied to single gene analysis of differential expression. Genes are ranked by magnitude of correlation with a class distinction, and the GSEA algorithm determines whether members of a gene set tend to occur towards the top or bottom of the list, and calculates an enrichment score. Microarray probe sequences were used to screen the RefSeq database using NCBI’s BLAST program to identify the human orthologue for each gene using an e value cut-off of 1.0E^-6^. Only probes that could be assigned a unique human gene symbol were taken forward for GSEA analysis. Gene sets that were significantly associated with birth weight or susceptibility classes were identified from the H (Hallmark), C2 (Curated), C5 (Gene Ontology), and C7 (Immunologic) collections of gene sets in the Molecular Signatures Database (MSigDB), which together comprise over 8000 individual gene sets [18]. The null distributions used to calculate the statistical significance of the enrichment scores were generated by permutation of class labels for the LBW v HBW contrast (>7 replicates/class) and of gene sets for the SUS v RES contrast (<7 replicates/class). Adjustment for multiple hypothesis testing was performed by determination of FDR. Enrichment scores with an FDR <0.25 were considered to be significant. Nominal (unadjusted) *P* values <0.01 were considered to exhibit a tendency for significance. Potentially important genes were identified by leading edge analysis of multiple significant gene sets within MSigDB collections. This method identifies those genes with large phenotype correlations, which contribute most to the enrichment score, in multiple gene sets within an MSigDB collection.

### Reverse Transcription-Quantitative Polymerase Chain Reaction (RT-qPCR)

RT-qPCR was carried out to validate the differential expression status of putative DEGs from the microarray experiments, and also to assess the expression of other genes of interest for this study, selected on the basis of known involvement in influenza response. Primer sequences were designed either from the pig genome sequence (Sscrofa 10.2 assembly) or from porcine ‘Refseq’ mRNA sequences (for unmapped genes) using the ‘Primer3’ program (bioinfo.ut.ee/primer3). Where possible, the forward primer was designed over an intron/exon boundary. A list of gene specific primer and RT-qPCR assay information that satisfy the ‘Minimal Information for the publication of Quantitative PCR Experiments’ (MIQE) requirements [19] is provided (S1 Appendix).

Reverse transcription reactions were carried out on samples from all 64 pigs (both individuals from each litter). One microgram of total RNA was reversed transcribed using a ‘High capacity cDNA reverse transcription’ kit (Life Technologies; Carlsbad, USA). PCR assays were performed in duplicate using 1 μl of a 1/25 dilution of each cDNA as template. Reactions were carried out using Kappa SYBR Fast Master Mix (Kappa Biosciences; Oslo, Norway) and 200 nmol of each primer in 20 μl volume on a 7900 HT Fast Real-Time PCR System (Life Technologies). A no-template negative control reaction was included for each gene. The PCR conditions were 95°C for 20 s, then 40 cycles of 95°C for 3 s and 60°C for 20 s. A melt curve analysis was carried out for each assay to validate its specificity. Also, PCR products were run on a 3% (w/v) agarose gel with a 50 bp ladder to check that the product size matched that predicted by the Primer3 software.

Quantification cycle (C_q_) values were determined using a manual ΔR_n_ threshold of 0.2. Reaction efficiencies were calculated for each assay from a standard curve generated from a 5-fold serial dilution series spanning 5 orders of magnitude. Expression data for DEGs of interest were normalized using values for the reference gene 18S rRNA, chosen for the stability of its expression across the sample set and a previous report of its reliability as a reference gene for RT-qPCR based on influenza-infected cells [20]. RT-qPCR expression data were analyzed using the Relative Expression Software Tool (REST) 2009 (http://www.gene-quantification.de/rest-2009.html) [21]. Normalized, relative expression ratios were calculated using the Pfaffl method [22] and statistical significance (*P*<0.05) was calculated by the randomization test in the software.

### DNA Methylation Analyses

Three genes that were differentially expressed in both the LBW v HBW and SUS v RES comparison, were selected for DNA methylation analyses: *ISG15*, *OAS1*, and *OAS2*. Sequences were obtained for the promoter region of each gene (designated as 1 Kb upstream of the transcriptional start site) from the pig genome sequence (S.scrofa 10.2 assembly), and for a CpG island in the first intron of *OAS2*. It was only possible to obtain 274 bp of promoter sequence for *OAS2* due to a gap in the current pig genome assembly. Primer pairs that work on bisulfite-converted DNA were then designed to span each region using MethPrimer (www.urogene.org/methprimer/). DNA samples (see section 2.3 for isolation protocol) were bisulfite-converted using the EZ DNA Methylation kit (Zymo Research) and fragments were amplified using the Complete PCR Reagent Set (Agenta Biosciences). The methylation percentage at each CpG dinucleotide was determined by mass spectrometry (Sequenom EpiTyper Assay). Mean values were calculated for each fragment and for each region as a whole for individual animals, and a comparison of the difference in % methylation between groups was performed using the Mann-Whitney U test in STATA 13 (Stata Corporation).

## Results

### Influenza Severity in Low and High Litter Birth Weight Phenotypes

Detailed phenotypic results were published previously [15], but pertinent details are presented herein to help visualize group differences in influenza severity. The birth weight of HBW and LBW groups were 1.61 ±0.07 kg and 1.29 ±0.17 kg respectively (*P*<0.05), and corresponded to Z-scores of 1.0 and -1.9 respectively (*P*<0.05). The mean growth rate during lactation was the same for both groups: 0.214 ±0.043 kg/day. Although no clinical parameter differed by group, the percentage of total lung affected by lesions was significantly higher in the HBW (14.2 ±9.2%) compared to the LBW (10.1 ±8.3%) group (*P* = 0.03). Similarly, microscopic lung lesion severity (*P* = 0.009) and influenza A immunoreactivity in middle lobes were also significantly higher in the HBW compared to LBW pigs (*P*<0.05). The virus concentration in lung tissue homogenates trended higher in HBW (3.5 ±1.4 log_10_ TCID_50_/ml in HBW; 3.0 ±1.5 log_10_ TCID_50_/ml in LBW; *P* = 0.09).

### Effect of Litter Birth Weight Phenotype on Gene Expression in the Lung of Influenza-infected Pigs

#### Differentially Expressed Genes in LBW v HBW Comparison

A total of 63 probes that correspond to 45 genes were identified as differentially expressed between the lung of HBW and LBW groups following microarray analysis (S2 Appendix.). Of these 45 genes, 29 were upregulated and 16 downregulated in LBW pigs respectively. Among the 29 upregulated genes were the T cell chemokines *CCL17* and *CCL25*, and two other genes expressed in lymphocytes: *CD3E* and *LEF1*. The downregulated genes included the three antiviral genes *ISG15*, *OAS1* and *OAS2*, and *CCL28*, a chemokine with antimicrobial activity.

#### Gene Set Enrichment Analysis in LBW v HBW Comparison

No gene sets were identified as being significantly associated with the LBW phenotype, although 24 gene sets exhibited a tendency for association. For the HBW phenotype, 8 gene sets had a significant association and a further 16 gene sets had a tendency for association (*P*<0.01) (S3 Appendix). The top 5 most enriched gene sets in LBW animals from each of the C2, C5, and C7 MSigDB collections that were enriched in LBW animals are shown in Table 1 (No H gene sets had a nominal P value <0.01). Cellular proliferation is a common theme to most of the C2 and C5 gene sets. Several cell cycle control genes belong to at least two C2 gene sets. These include the anti-apoptotic factor *BIRC5* and mitosis-promoting factors *CDC25C*, *CCNB1*, and *CDK1*. The translation elongation factors *EIF2B2* and *EIF2B4* belong to four C5 gene sets. The top 5 C7 gene sets include three that are enriched in T cells compared to other immune cell types. Several important T cell genes belong to at least two gene sets. These include *DGKA* and *ICOS*, that both have a role in T cell receptor (TCR) signaling.

**Table 1:** Top 5 Gene Sets Enriched in LBW Pigs

MSigDB Collection	Gene Set Name	Gene Set Identifier	Normalized Enrichment Score*
**C2 (Curated Gene Sets)**	Dairkee Cancer Prone Response BPA	M8655	1.94
	Chemello Soleus vs EDL Myofibers UP	M3001	1.94
	Biocarta G2 Pathway	M8560	1.87
	Park HSC and Multipotent Progenitors	M1456	1.73
	Whitfield Cell Cycle Literature	M2066	1.70
**C5 (GO Gene Sets)**	Response to Hormone Stimulus	M13987	1.88
	Cellular Response to Stimulus	M4602	1.85
	DNA Integrity Checkpoint	M16357	1.79
	Reproductive Process	M15843	1.56
	Reproduction	M5029	1.52
**C7 (Immunologic Signatures Gene Set)**	GSE22886 Naïve CD4 T cell vs NK cell UP	M4423	1.81
	GSE24634 Naïve CD4 T cell vs Day 5 IL4 Treg conv UP	M4588	1.69
	GSE1448 CTRL vs Anti Valpha2 DP Thymocyte UP	M3425	1.68
	GSE27786 LSK vs NKT cell UP	M4756	1.65
	GSE22886 Naïve CD8 T cell vs NK cell UP	M4418	1.64

* Have an associated nominal *P* value of <0.01

**Fig 1:**
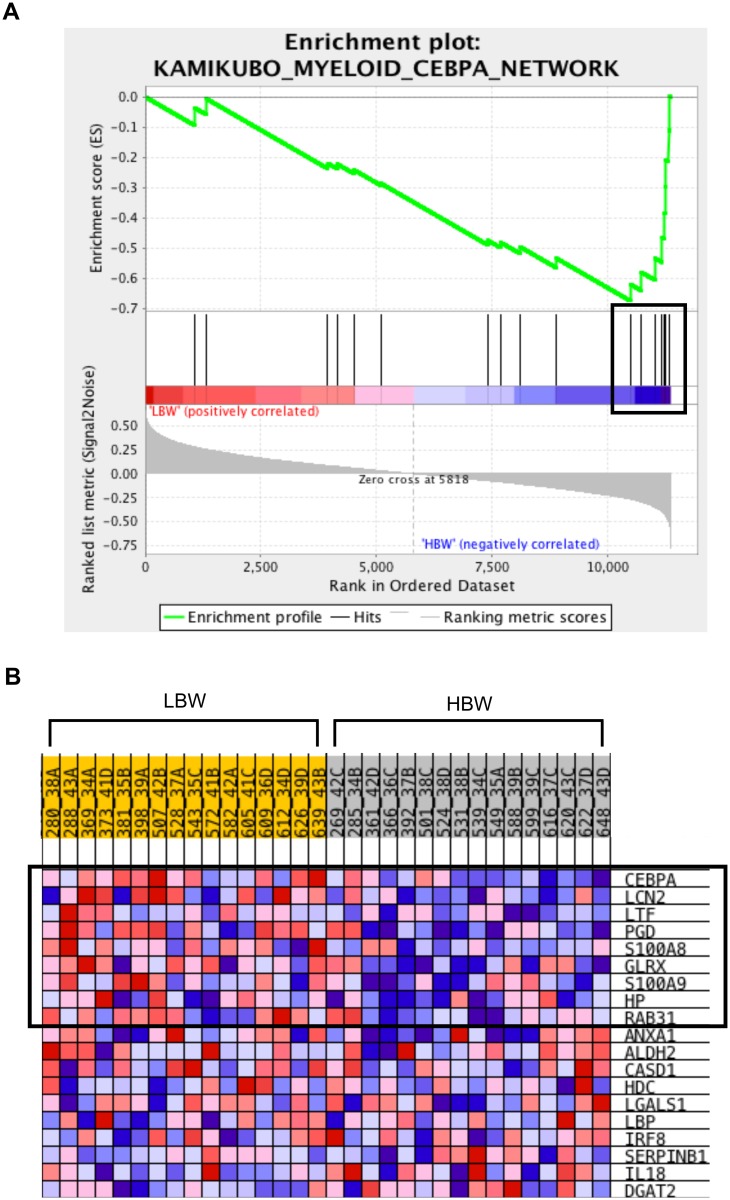
Enrichment of a Myeloid CEBPA Network in HBW Pigs. Enrichment result for the ‘Kamikubo Myeloid CEBPA Network’ gene set from the Gene Set Enrichment Analysis of transcriptional data from low and high birth weight pigs (LBW and HBW). (A) Enrichment plot used to calculate the enrichment score for that gene set. The score is calculated by walking down a list of genes ranked by their correlation with the LBW phenotype, increasing a running-sum statistic when a gene in that gene set is encountered (each black vertical line underneath the enrichment plot) and decreasing it when a gene that isn’t in the gene set is encountered. The enrichment score is the maximum deviation from zero encountered in the walk. (B) Heat map of correlation values for all individual genes within the gene set. Color and shade indicate direction and magnitude of correlation; red indicates positive correlation with LBW (negative correlation with HBW) and blue indicates negative correlation with LBW (positive correlation with HBW). Darker shades correspond to correlation values of greater magnitude. Those genes that contribute most to the enrichment score, the leading edge subset of genes, are outlined by a box in both the enrichment plot and the heatmap.

Table 2 contains the top 5 most enriched gene sets for the C2, C5, and C7 collections in HBW pigs (No H gene sets had a nominal *P* value of <0.01). Three of the C2 gene sets contain genes activated in response to immune signaling pathways: interleukin-22, JNK, and myeloid cell CEBPA signaling. The enrichment result for the CEBPA signaling pathway is shown in Fig 1. Genes that appear in at least two of these gene sets include complement component *C3* and factor *CFB*, and monocyte/macrophage cell membrane proteins *CD14* and *CD74*. Only two GO terms were enriched in HBW pigs, ‘Peptidyl tyrosine modification’ and ‘Peptidyl tyrosine phosphorylation’. Two tyrosine kinases appear in both gene sets, *EGFR* and *ABL2*. Other genes of interest include two antiviral cytokines, *IFNL1* and *IL12A*, the integrin *ITGB2*, which is the receptor for complement component 3, and coagulation factor *F2*. Four of the top 5 immunological signature gene sets correspond to transcriptional analyses using cells of the myeloid lineage, and the three most enriched gene sets contain genes that are more highly expressed in these cells (monocytes or dendritic cells) than lymphocytes (cytotoxic or helper T cells or B cells lymphocytes). Eleven genes appear in three of these gene sets. They include the chemokine receptor *CCR1*, which encodes the receptor for macrophage inflammatory protein 1 alpha and monocyte chemoattractant protein 3. Two phagolysosomal enzyme genes, *CTSB* and *CTSH*, and two pattern recognition receptor (PRR) pathway genes, *CLEC7A* and *LY96*, were also among these genes.

**Table 2:** Top 5 Gene Sets Enriched in HBW Pigs.

MSigDB Collection	Gene Set Name	Gene Set Identifier	Normalized Enrichment Score*
**C2 (Curated Gene Sets)**	Zheng IL22 Signaling UP^a^	M1800	-2.03
	Han JNK Signaling DN^a^	M1655	-1.96
	Kamikubo Myeloid CEBPA Network^a^	M2092	-1.93
	Tsunoda Cisplatin Resistance DN^a^	M5014	-1.90
	KEGG Sphingolipid Metabolism	M15955	-1.88
**C5 (GO Gene Sets)** ^b^	Peptidyl Tyrosine Modification^a^	M19329	-2.09
	Peptidyl Tyrosine Phosphorylation^a^	M6727	-1.96
**C7 (Immunologic Signatures Gene Set)**	GSE22886 Naïve B Cell vs Monocyte DN^a^	M4486	-1.90
	GSE22886 Naïve CD4 T cell vs DC DN^a^	M4503	-1.85
	GSE22886 Naïve CD8 T cell vs DC DN	M4476	-1.75
	GSE20715 0h v 24h Ozone Lung DN	M4367	-1.69
	GSE36392 Type 2 Myeloid vs Mac IL25 Treated Lung UP	M5273	-1.58

*Nominal P value <0.01. Negative value as enrichment score calculated with respect to LBW phenotype

^a^FDR<0.25

^b^Only two C5 gene sets had a nominal *P* value <0.01

#### RT-qPCR for LBW v HBW Comparison

**Fig 2:**
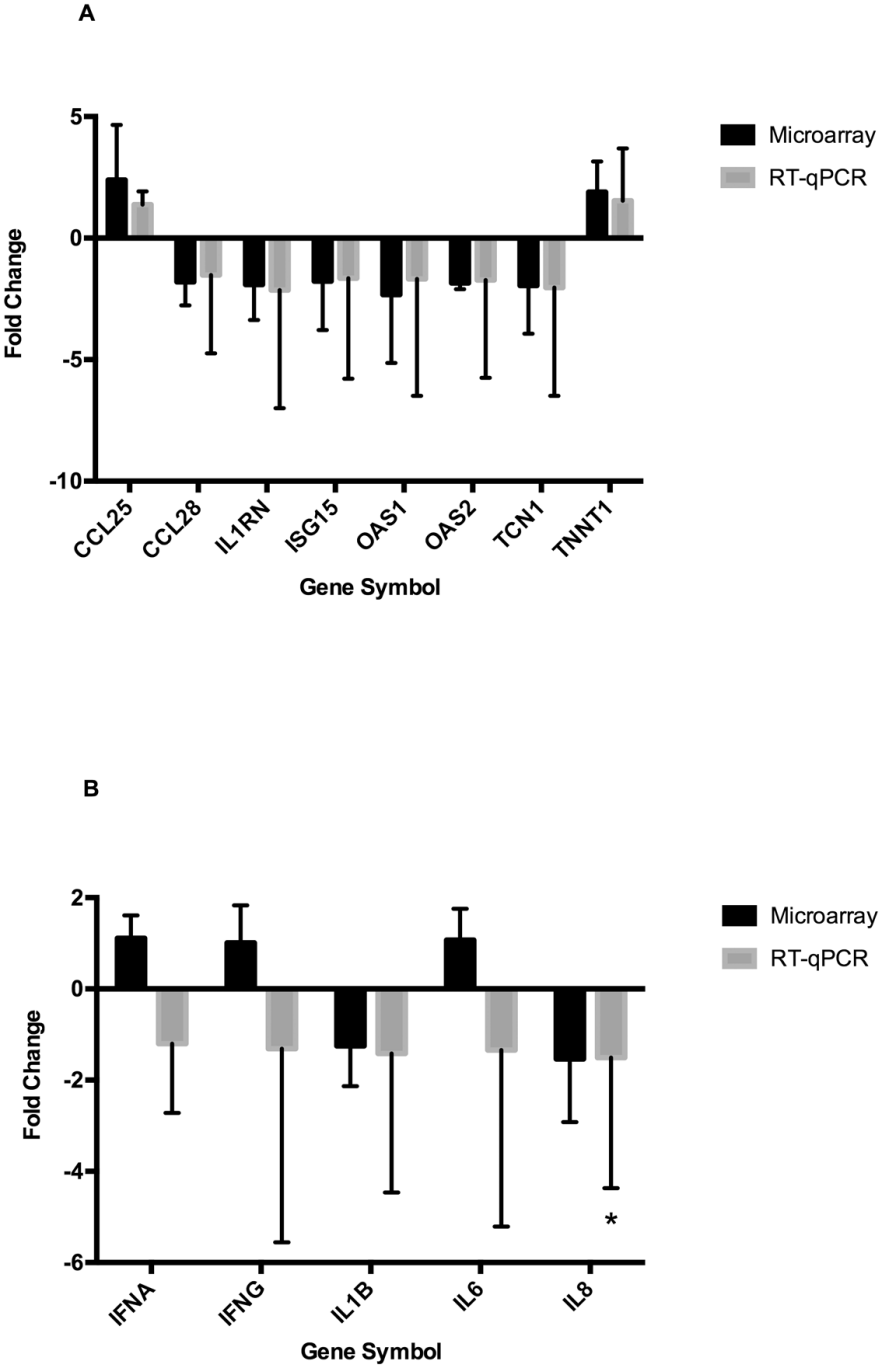
Expression Profiling of Individual Genes in Low and High Litter Mean Birth Weight Pig Groups. (A) Fold change in low (LBW) relative to high birth weight (HBW) pigs of genes identified by microarray and confirmed by RT-qPCR as being differentially expressed. All fold changes shown are statistically significant (*P*<0.05). (B) Fold change in LBW relative to HBW pigs of a panel of cytokine genes as measured by microarray and RT-qPCR. Statistically significant fold changes are denoted with an asterisk (* *P*<0.05).

The expression status of 14 genes of biological interest identified as differentially expressed by microarray was verified by RT-qPCR. The direction and significance of differential expression was confirmed for 8 genes (*CCL25*, *CCL28*, *IL1RN*, *ISG15*, *OAS1*, *OAS2*, *TCN1*, and *TNNT1*), of which 6 genes were less highly expressed in the LBW compared to the HBW group (Fig 2A). Six of the eight validated DEGs are annotated with the Gene Ontology (GO) term ‘immune response’ (GO:0006955): *CCL25*, *CCL28*, *IL1RN*, *ISG15*, *OAS1*, and *OAS2*. Three of these genes, all less highly expressed in the LBW group, were also annotated with the term ‘defense response to virus’: *ISG15*, *OAS1*, and *OAS2*. Further RT-qPCR assays were performed for a panel of cytokine genes previously reported as being involved in the host response to influenza infection: *IFNA*, *IFNG*, *IL1B*, *IL6*, and *IL8*. The *IL8* gene had significantly lower expression in the LBW than HBW group (*P*<0.05), but no expression difference between groups were observed for the other cytokines (Fig 2B).

### Comparison of Gene Expression in the Lung of Pigs Exhibiting Divergent Severity of Lung Influenza Pathology

#### Differentially Expressed Genes in SUS v RES Comparison

A total of 131 probes that correspond to 91 genes were identified as being differentially expressed in the lung of ‘SUS’ and ‘RES’ pigs following influenza infection (S2 Appendix). Of these 91 genes, 65 were more highly expressed in the SUS group and 26 were less highly expressed. Prominent among the genes upregulated in the SUS group (downregulated in the RES) were those that participate in the inflammatory response. The most highly up-regulated gene was *TCN1*, whose protein is a major constituent of the secondary granules of neutrophils. A number of pro-inflammatory cytokine genes were also upregulated in SUS, including *CXCL2* and *CCL2*, chemotactic cytokines for neutrophils and macrophages respectively, and *IL6*. In addition, genes that function in other inflammation-associated processes such as complement activation (*C1R*, *C1S*, *SERPING1*), coagulation (*SERPINE1*, *ANXA8*, *THBS1*), and tissue re-modeling (*TIMP1*, *TNFRSF12A*) were also upregulated. Several genes involved in the negative regulation of inflammation were also upregulated, such as the interleukin-1 beta antagonist *IL1RN*, and *SFN*, a negative regulator of several Toll-like Receptor (TLR) signaling cascades [23]. Finally, the antiviral gene *OASL* was found to be upregulated in SUS animals.

Among the 51 genes that were downregulated in SUS group (upregulated in RES) were several components of the electron transport chain: the mitochondrial-encoded cytochrome B (*CYTB*) and NADH dehydrogenase subunits 4 and 5 (*ND4* and *ND5*). In addition, several genes whose products are components of the protein translation machinery were also upregulated: *RPL5*, *RPL11*, and *EEF1A1*. Only two genes with obvious immune functions were upregulated in RES animals, and both are involved in the allergic response: the immunoglobulin epsilon receptor gene *FCER1A* was the most up-regulated gene, and *ENPP3* encodes an enzyme expressed in basophils and mast cells.

#### Gene Set Enrichment Analysis of SUS v RES Comparison

Gene set enrichment analysis identified 531 gene sets that were significantly enriched in SUS pigs and 172 in RES pigs (S3 Appendix). Selected gene sets of interest from the top 25 in each MSigDB collection (H, C2, C5, and C7) are shown in Table 3. Many of the most significant Hallmark (H) gene sets relate to innate immunity and pro-inflammatory signaling. These are ‘Interferon gamma response’, ‘Inflammatory Response’, ‘Interferon alpha response’, ‘TNF-α signaling via NF-κB’, ‘IL6 JAK-STAT3 signaling’, and ‘Complement’. Three genes belong to five of these gene sets: the monocyte chemokines *CXCL10* and *CXCL11*, and the pro-inflammatory cytokine *IL6*. The two interferon-signaling pathways share a block of genes that function in the innate immune response to viral infection, which includes the DEG *OASL* as well as *IRF7*, *STAT2*, *DDX58*, *ISG15*, *USP18*, and *MX1*. Inflammatory response genes include the pro-inflammatory cytokines *CCL2*, *IL1B*, *IL6*, and *IL8*, Pattern Recognition Receptors *TLR1*, *TLR2*, *TLR3*, and *CLEC5A*, and intracellular signaling molecules *NFKBIA*, *IRAK2*, *RIPK2*, and *RASGRP1*.

**Table 3:** Selected Gene Sets Enriched in SUS Pigs.

MSigDB Collection	Gene Set Name	Gene Set Identifier	Normalized Enrichment Score*
**H (Hallmark)**	Interferon Gamma Response	M5913	2.62
	Inflammatory Response	M5932	2.43
	Interferon Alpha Response	M5911	2.42
	TNFA Signaling Via NFKB	M5890	2.34
	IL6 JAK-STAT3 Signaling	M5897	2.05
	Complement	M5921	1.75
**C2 (Curated Gene Sets)**	Sana TNF Signaling UP	M17466	2.75
	Hecker IFNB1 Targets	M3010	2.57
	Reactome Interferon Alpha Beta Signaling	M973	2.31
	Browne Interferon Responsive Genes	M9221	2.27
	Der IFN Alpha Response UP	M3652	2.20
	Radaeva Response to IFNA1 UP	M13763	2.17
	Phong TNF Response Via P38 Partial	M2502	2.16
**C5 (GO Gene Sets)**	Chemokine Receptor Binding	M5006	2.02
	G Protein Coupled Receptor Binding	M14760	1.97
	Chemokine Activity	M14051	1.92
	Inflammatory Response	M10617	1.89
	Response to Wounding	M5634	1.84
	Cytokine Activity	M14581	1.77
	Defense Response	M3458	1.61
**C7 (Immunologic Gene Sets)**	GSE18791 CTRL vs Newcastle Virus DC 8h DN	M4267	2.31
	GSE18791 CTRL vs Newcastle Virus DC 10h DN	M4269	2.23
	GSE18791 Unstim vs Newcastle Virus DC 6h DN	M4292	2.20
	GSE14000 Unstim vs 4h LPS DC Translated RNA DN	M3338	2.18
	GSE2706 Unstim vs 2h LPS and R848 DC DN	M4703	2.12
	GSE18791 Unstim vs Newcastle Virus DC 10h DN	M4294	2.12
	GSE2706 Unstim vs 2h LPS DC DN	M4696	2.06
	GSE18791 CTRL vs Newcastle Virus DC 4h DN	M4261	2.05

*FDR<0.25

**Fig 3:**
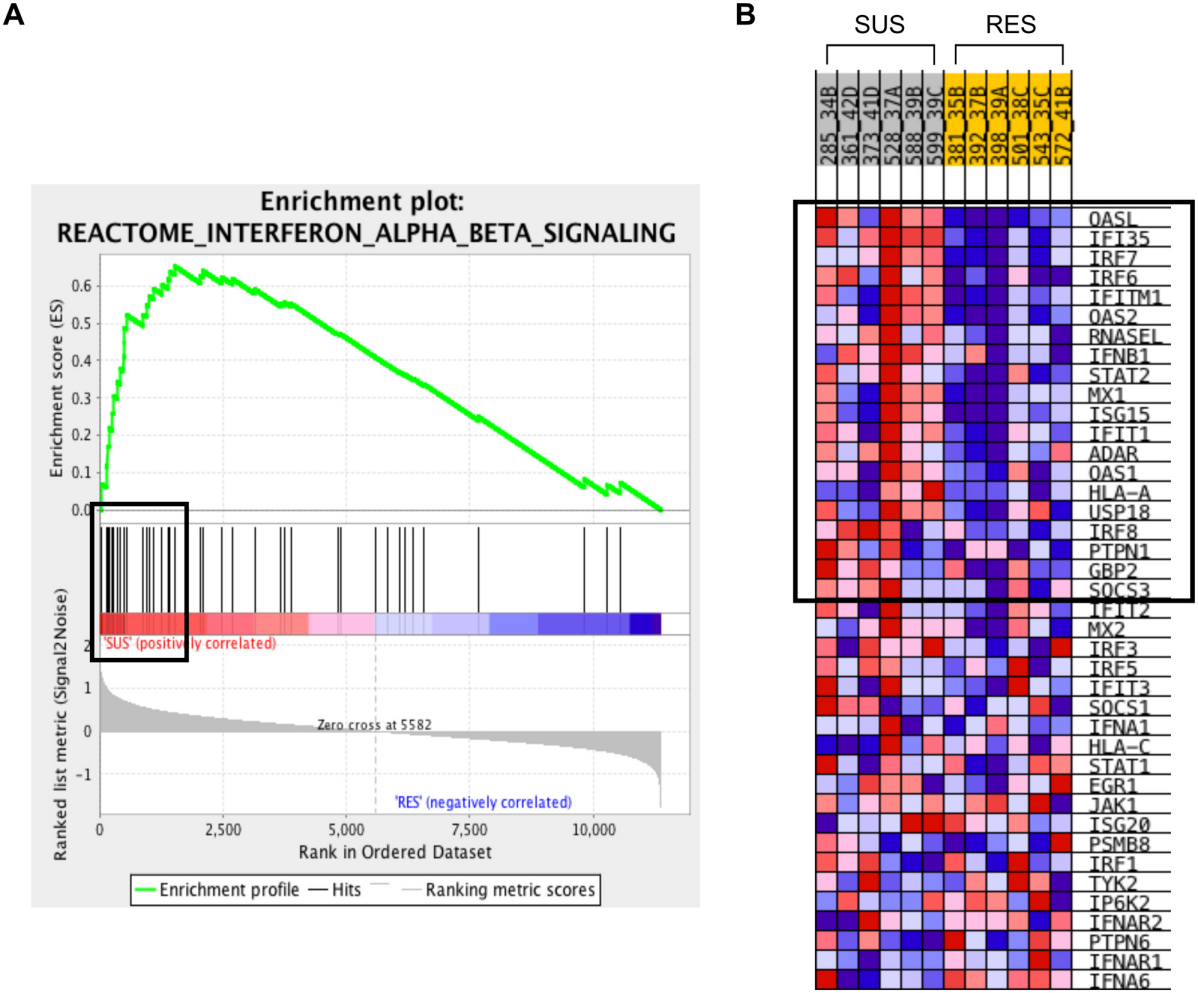
Enrichment of a Type I Interferon Gene Set in SUS Pigs. Enrichment result for the ‘Reactome Interferon Alpha Beta Signaling’ gene set from the Gene Set Enrichment Analysis of transcriptional data from pigs that are susceptible (SUS) or resistant (RES) to influenza pathology. (A) Enrichment plot used to calculate the enrichment score for that gene set. The score is calculated by walking down a list of genes ranked by their correlation with the LBW phenotype, increasing a running-sum statistic when a gene in that gene set is encountered (each black vertical line underneath the enrichment plot) and decreasing it when a gene that isn’t in the gene set is encountered. The enrichment score is the maximum deviation from zero encountered in the walk. (B) Heat map of correlation values for all individual genes within the gene set. Color and shade indicate direction and magnitude of correlation; red indicates positive correlation with SUS (negative correlation with RES) and blue indicates negative correlation with SUS (positive correlation with RES). Darker shades correspond to correlation values of greater magnitude. Those genes that contribute most to the enrichment score, the leading edge subset of genes, are outlined by a box in both the enrichment plot and the heatmap.

Over 300 C2 gene sets were enriched in SUS pigs, encompassing a wide variety of biological processes. Nevertheless, examination of the 25 most significant sets found that many of them shared common functional attributes. They include five type I interferon signaling related gene sets. The enrichment result for one of these gene sets is shown in Fig 3. Genes common to all 5 sets included the previously mentioned *OAS1*, *MX1*, and *ISG15* as well as the interferon-stimulated gene *IFI35*. Two TNF-α, signaling related gene sets were also enriched.

The two most significant C5 gene sets enriched in SUS pigs were the GO terms ‘Chemokine receptor signaling’ and ‘G protein coupled receptor signaling’. The same genes are annotated to both terms and are the aforementioned *CCL2*, *CXCL2*, *CXCL10*, *CXCL11*, and *IL8*, as well as *CCR2*, the receptor for monocyte chemotactic protein 1, three IFN-γ activated chemokines *CXCL9* and *CXCL12*, and *CCL20*, and the granulocyte chemoattractant *CXCL6*. The GO terms ‘Inflammatory response’ and ‘Response to Wounding’ were also among those enriched in SUS pigs. In addition to many pro-inflammatory signaling genes, some genes with notable anti-inflammatory effects, *ANXA1*, *IL10RB1*, and *TNFAIP6*, contributed to the core enrichment score of these gene sets.

The top 25 C7 immunological signature gene sets associated with the SUS phenotype contained 8 collections of genes that were upregulated in stimulated monocytes, macrophages or dendritic cells compared to unstimulated controls. Most of the treatments in these experiments were exposure of cells to virus, and antiviral, type-I interferon responsive genes were found in multiple gene sets; the most common being *ISG15*, *OASL*, and *IFIH1* in all 8 gene sets.

In total, 172 gene sets were significantly enriched in RES pigs (S3 Appendix). Selected gene sets from the Top 25 H, C2, C5, and C7 gene sets are shown in Table 4. Only one H gene set, ‘Spermatogenesis’, was enriched in ‘RES’ animals. Spermatogenesis is obviously not a biological process associated with lung tissue, but some of the genes annotated with that term do function in lymphocyte biology, such as *MLLT10*, *SHE*, *SNAP91*, and *IL12RB2*. Protein synthesis is the biological process associated with the most enriched gene sets in both the C2 and C5 MSigDB collections. Thirty-four genes contribute to the core enrichment score of the six C2 gene sets that relate to protein synthesis, all of which are structural components of the small or large subunits of the eukaryotic ribosome. Interestingly, one of these pathways relates to influenza infection: ‘Influenza Viral RNA Transcription and Regulation’. Two other GO terms enriched in RES pigs relate to the extracellular matrix. Genes associated with these terms encode extracellular matrix components (ECMs) such as collagens COL15A1, COL4A5, and COL5A2 and other structural components of collagen fibril matrices such as FBN1 and LUM. Forty C7 immunological signature gene sets were enriched in RES pigs. Four out of the Top 25 sets contain genes that are more highly expressed in lymphocytes than cells of the myeloid lineage. Genes common to three or more of these sets include TCR complex genes *CD3G*, *CD3E*, *CD3G*, and CD247, membrane receptor *BCL2* and the transcription factor *LEF1*.

**Table 4:** Selected Gene Sets Enriched in RES Pigs.

MSigDB Collection	Gene Set Name	Gene Set Identifier	Normalized Enrichment Score*
**H (Hallmark)**	Spermatogenesis	M5951	-1.52
**C2 (Curated Gene Sets)**	KEGG Ribosome	M189	-2.57
	Reactome Peptide Chain Elongation	M13642	-2.51
	Reactome SRP Dependent Cotranslational Protein Targeting to Membrane	M567	-2.43
	Reactome 3 prime UTR Mediated Translational Regulation	M781	-2.39
	Reactome Influenza Viral RNA Transcription and Replication	M7636	-2.32
	Reactome Translation	M8229	-2.29
**C5 (GO Gene Sets)**	Structural Constituent of Ribosome	M13114	-2.26
	Structural Molecule Activity	M14147	-1.87
	Proteinaceous Extracellular Matrix	M15654	-1.82
	Extracellular Matrix	M18403	-1.78
	Translational Initiation	M11328	-1.71
**C7 (Immunologic Gene Sets)**	GSE22886 Naïve T cell vs DC UP	M4475	-1.95
	GSE22886 Naïve CD8 T cell vs Monocyte UP	M4494	-1.75
	GSE22886 Naïve CD4 T cell vs Monocyte UP	M4504	-1.67
	GSE22886 Naïve CD8 T cell vs DC UP	M4490	-1.55

*FDR<0.25

#### RT-qPCR of SUS v RES Comparison

**Fig 4:**
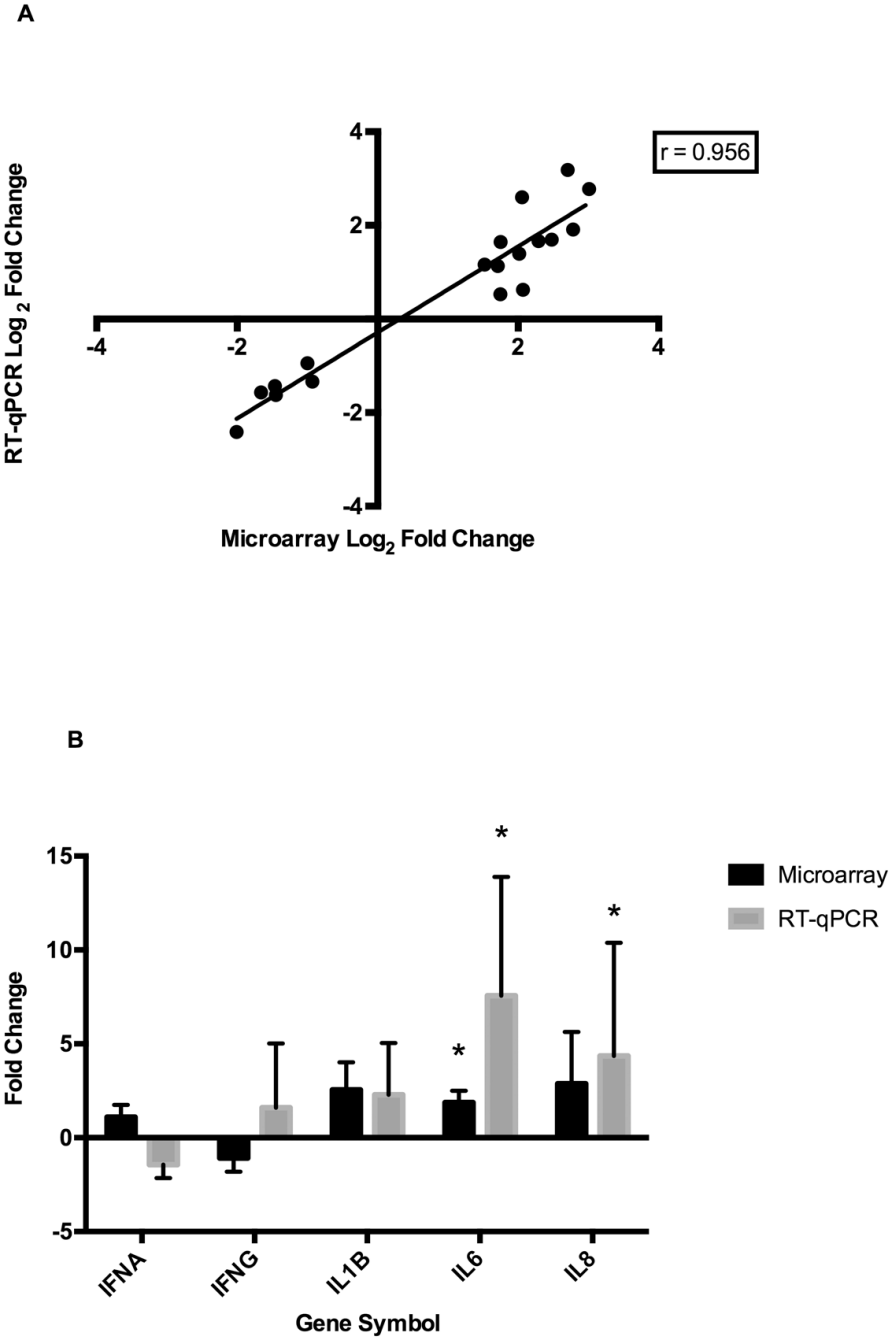
Expression Profiling of Individual Genes in Pigs with Divergent Severity of Influenza Pathology. (A) Scatter plot of fold changes for 18 genes in the ‘Susceptible’ (SUS) relative to ‘Resilient’ (RES) group of pigs measured by two different methods: microarray hybridization (X-axis) and RT-qPCR (Y-axis). The Pearson Correlation coefficient of the two variables is shown. The 18 genes were identified as DEGs by microarray and the expression pattern was confirmed for 15/18 genes (*P*<0.05). Overall the two measures of expression differences are highly correlated, indicating that there is generally good agreement between fold changes of DEGs measured by each method. (B) Fold change in SUS relative to RES pigs of a panel of cytokine genes as measured by microarray and RT-qPCR. Statistically significant fold changes are denoted with an asterix (*P<0.05).

The expression status of 18 genes, identified as differentially expressed between SUS and RES pigs by microarray, was validated by RT-qPCR. The DEG status of the following 15 genes was confirmed: *APOA1*, *C1R*, *C1S*, *CCL2*, *CP*, *CRABP1*, *CXCL2*, *FCER1A*, *IL1RN*, *IVNSABP1*, *ND4*, *ND5*, *OASL*, *SFN*, and *TCN1*. For the other 3 genes (*GBP15*, *SERPINE1*, and *SERPING1*), the direction of change in gene expression was consistent with what was observed by microarray, but results were not statistically significant. The Pearson correlation coefficient for gene expression changes measured by microarray and RT-qPCR for this set of genes was 0.956 (Fig 4A) Additional RT-qPCR assays were performed for the same panel of cytokine genes described for the LBW v HBW contrast: *IFNA*, *IFNG*, *IL1B*, *IL6*, and *IL8*. The *IL6* and *IL8* genes had significantly higher expression in the SUS than RES group (*P*<0.05), but no significant expression difference between groups was observed for the other cytokines (Fig 4B). Finally, RT-qPCR data was also available for those genes tested in the validation experiments for the LBW v HBW comparison (as the SUS v RES comparison uses a subset of these animals). Three genes from the LBW v HBW comparison that were not identified as DEG by microarray for the SUS v RES comparison were nevertheless found to be differentially expressed for this comparison as well when tested by RT-qPCR. These were the cytokine gene *CCL28*, and the two innate antiviral genes *OAS1* and *OAS2*.

### Epigenetic analysis of innate antiviral genes

**Fig 5:**
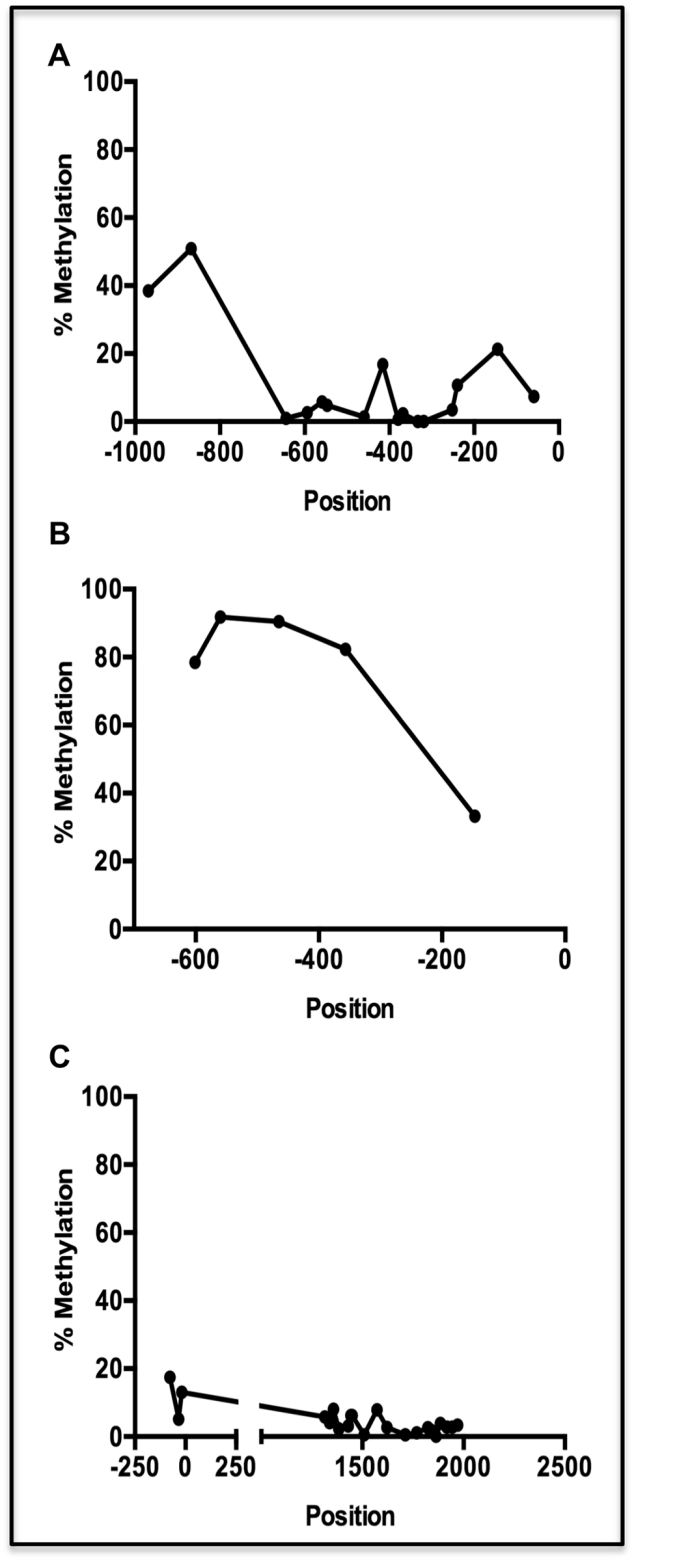
DNA Methylation at CpG Dinucleotides Across the Promoter Regions of Three Antiviral Genes. Mean DNA methylation at individual CpG dinucleotide sites along the promoter regions of three antiviral genes that are differentially expressed in both low and high birth weight groups, and resilient and susceptible groups of pigs: *ISG15* (A), *OAS1* (B), or *OAS2* (C). The extent of methylation in a CpG island in intron 1 of *OAS2* (C) is also shown. Positions are given as base pairs up (-) or down (+) stream of the putative transcriptional start site (TSS).

**Fig 6:**
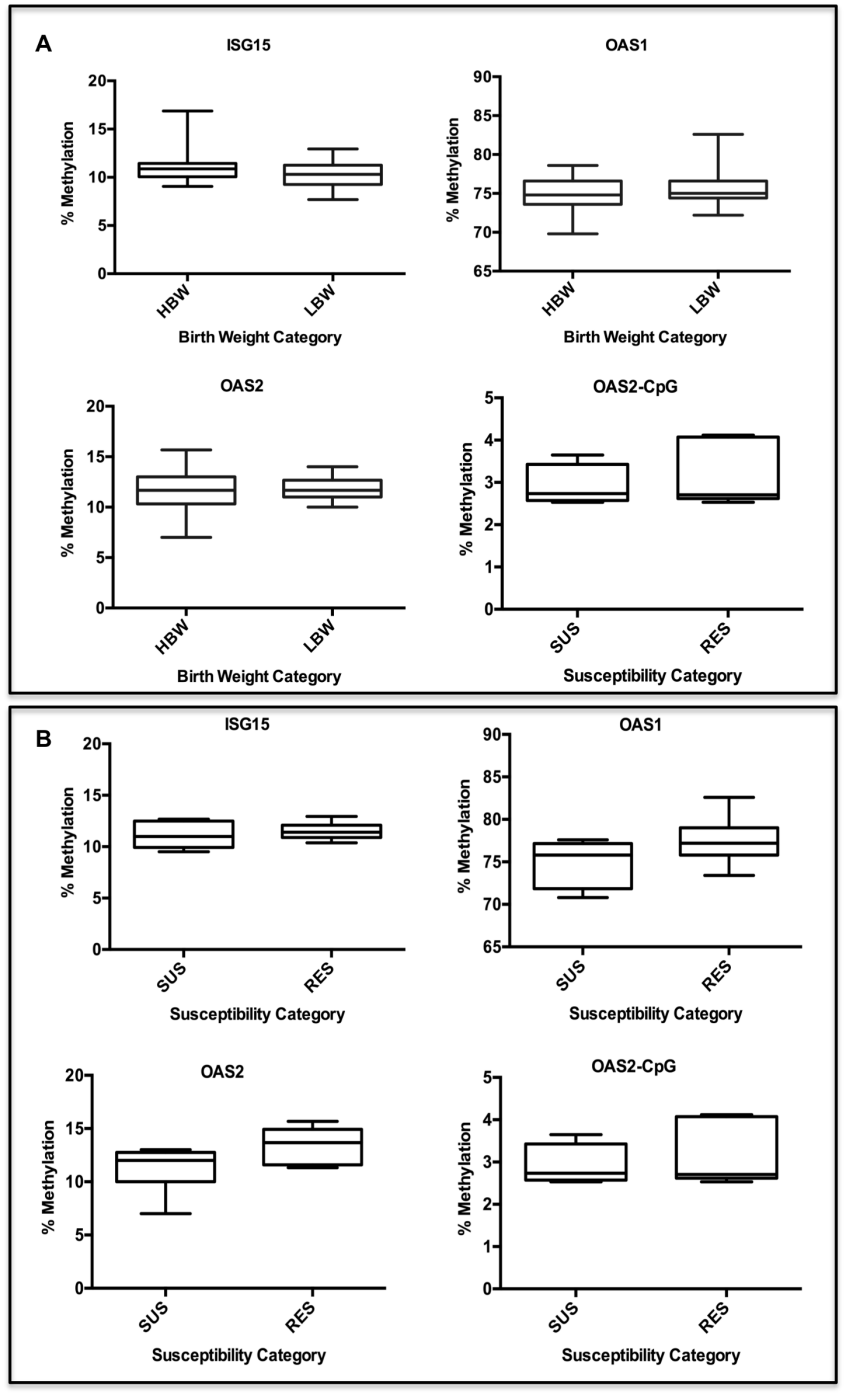
Overall DNA Methylation Within Promoter Regions of Three Antiviral genes. Comparison of extent of CpG methylation (mean of all CpG sites for pigs in each group) between LBW and HBW groups (A) and SUS and RES groups (B) within the promoter regions of *ISG15*, *OAS1*, and *OAS2*, and a CpG island in intron 1 of *OAS2*. There were no statistically significant differences between groups across any of the regions of interest.

One possible cause for the differential expression of genes in the LBW and HBW groups is the epigenetic modification of gene regulation instigated by prenatal programming. To test this, the extent of DNA methylation (%) within the promoter regions of three differentially expressed antiviral genes: *ISG15*, *OAS1*, and *OAS2*, and a putative CpG island within the first intron of *OAS2*, was investigated. The extent of CpG methylation in the promoter regions varied between genes. The promoters of *ISG15* and *OAS2* had relatively low levels of methylation, 10.48 ±3.71%) and 11.87 ±3.62%) respectively, whereas that of *OAS1* was much more methylated (75.25 ±10.80%). The % methylation at the putative CpG island in intron 1 of *OAS2* was very low (3.47 ±0.54%), and confirms that this region of the genome is relatively unmethylated. For each gene, the % methylation within the promoter region is lower in the vicinity of the putative transcriptional start site (TSS) (Fig 5A). However, the degree of DNA methylation was generally very consistent between individual pigs for a given gene. None of the regions taken as a whole were differentially methylated between either the HBW or LBW groups (Fig 5B) or the RES and SUS groups (Fig 5C). Two individual CpG sites were found to be less methylated in SUS than RES animals: one site 357 bp upstream of the *OAS1* TSS (RES: 86.50 ±0.43%; SUS 80.83 ±1.74%; *P*<0.01) and another site 34 bp upstream of the *OAS2* TSS (RES: 6.33 ±0.49%; SUS: 4.33 ±0.33%; *P*<0.05). However, no significant differences between HBW and LBW animals were identified at any of the individual CpG-containing fragments spanning the regions of interest (Fig 6).

## Discussion

Previous epidemiological studies and animal experiments have shown that gestational insults such as stress and undernutrition can have a detrimental effect on post-natal immune function and susceptibility to infectious diseases. The epigenetic modification of gene transcription has been proposed as the mechanism responsible for mediating the effects of these environmental factors, but very few studies have looked for the causative changes at the DNA and RNA level that underpin these effects on immune function. The principal aim of this study was to investigate whether there was any evidence for such ‘prenatal programming’ effects on the expression and epigenetic regulation of genes in commercial pig populations, using litter mean birth weight as a proxy for intrauterine growth restriction and severity of influenza pathology as our response trait of interest. The second aim of the study was to compare the transcriptome of pigs classified as ‘resilient’ or ‘susceptible’ to influenza on the basis of lung pathology, and irrespective of birth weight phenotype. This was done to help inform the results from the main birth weight analysis, and to obtain a greater understanding of the molecular pathways involved in the pathology of swine influenza.

Only minor differences were observed in the transcriptome of HBW and LBW groups of pigs in this study, in contrast to the larger differences between the RES and SUS groups, and the results should be interpreted with caution due to the less stringent significance thresholds adopted. Nevertheless, the expression profile of HBW pigs resembled that of SUS pigs to an extent, and likewise gene expression in LBW pigs had similarities with that of RES pigs. The HBW and SUS groups shared a higher expression of antiviral and leukocyte genes from cells of the myeloid lineage, including some pro-inflammatory cytokines. In LBW and RES pigs there was an enrichment of T cell associated genes. These results are consistent with the experiment dataset as a whole, in which the LBW group were not immunologically compromised or more susceptible to influenza infection than HBW pigs, contrary to our original hypothesis [15]. In fact, viral loads trended lower, and pathological lesion scores were significantly lower, in the lung lobes of LBW than HBW pigs.

The type I interferon pathway is central to the innate immune response to viral infection in mammals [24]. Three interferon stimulated genes (ISGs) from this pathway were confirmed as having lower expression in LBW pigs by RT-qPCR: *ISG15*, *OAS1*, and *OAS2*. ISG15 is an ubiquitin-like modifier that can bind to and modify both host and viral proteins and antagonize virus replication [25]. The OAS protein family are enzymes that catalyze the formation of 2`-5`oligoadenylate from ATP upon binding viral double stranded RNA, which in turn causes the dimerization and activation of RNase L: a potent endonuclease for the degradation of viral RNA [26]. Both ISG15 and the OAS-RNase L system have been shown to reduce susceptibility to influenza in experimental models [27,28]. However, given the transcriptional similarities between LBW and RES pigs the most probable explanation for the lower expression of these genes is as a consequence of lower viral load rather than an indication of an increased susceptibility to infection. The interferon response pathway is directly activated by a number of cellular pattern recognition receptors for RNA viruses, such as TLR3, TLR7, and RIG-I [29], and so the expression ISGs in infected cells is positively associated with the amount of viral RNA in the cell.

Cells of the myeloid lineage also play important roles in the host response to influenza, in particular alveolar macrophages and dendritic cells. Alveolar macrophages are the predominant immune cell type in the lung under normal conditions, while dendritic cells are located in smaller numbers throughout the respiratory tract. Following activation by influenza virus, both cell types phagocytose influenza virions and infected, apoptotic epithelial cells, and produce a number of pro-inflammatory cytokines and chemokines that recruit and activate additional monocytes from local blood vessels. *CCR1*, whose expression was found to correlate with HBW phenotype by GSEA, is expressed on monocytes and is the receptor for the pro-inflammatory chemokines MCP3 and CCL3 (MIP1-α) [30]. Indicators of phagocytosis, such as cathepsins *CTSB* and *CTSH* were also identified. Phagocytosis is clearly important in limiting the spread of virus in the lungs during the early stages of influenza infection, but prolonged release of pro-inflammatory cytokines such as TNF-α results in excessive inflammation and pulmonary damage [31,32].

This study did not find any evidence for the epigenetic alteration of gene expression as a consequence of prenatal programming of birth weight phenotype. None of the promoter regions of the three ISGs downregulated in LBW pigs were differentially methylated. In addition, it seems unlikely that their lower expression is due to epigenetic silencing of their upstream regulators. The promoter regions of their regulators, type I interferons *IFNA* and *IFNB*, were not screened as neither of these genes was differentially expressed between birth weight groups. It is possible other non-tested DEGs were epigenetically regulated, or that epigenetic mechanisms such as histone acetylation and microRNA expression were responsible for the gene expression differences we found. The epigenetic alteration of gene expression in response to a low-protein nutritional model of prenatal programming has been demonstrated in rat [33,34]. It has also been observed in the neonatal programming of the rat HPA axis by maternal behavior [35,36]. In these studies, the expression of key genes was altered as a consequence of methylation status at specific CpG dinucleotides within their promoters. The epigenetic regulation of immune and inflammatory gene transcription is evident in many biological processes, such as Th1/Th2 cell development, macrophage differentiation, and tolerance of commensal bacteria [37], but no firm link to prenatal programming has yet been established.

Several studies in swine have demonstrated some long-term detrimental effects of low birth weight on glucose tolerance, cardiovascular function, HPA axis function, muscle development, and lean muscle growth [12,14,38–40]. Few studies have investigated the effect of low birth weight on the swine immune system, but Zhong *et al*. reported that IUGR piglets had a reduced immune competence in comparison to their normal weight littermates [41]. However, individuals in that study were >2 SD below the population mean, a traditional classification of IUGR that largely identifies “runt” pigs. Our study was interested in low birth weight as a litter characteristic, with a requirement that the mean litter birth weight be a minimum of >0.7 SD above or below the population mean after controlling for parity of dam and litter size. The individual animals selected for influenza inoculation had birth weights close to the mean for the litter, purposefully removing true runt pigs from consideration. Consequently, the limitations on growth imposed during gestation in our study were less severe, which, together with the different outcome variables studied (lymphocyte proliferation in their study and response to infection in ours), may contribute to the difference in results. Nevertheless, the wide disparity in mean Z-scores of the HBW and LBW groups, along with the fact that Z-score values did not overlap between groups, demonstrates that the piglets of each group did experience very different uterine environments.

Other mechanisms of prenatal programming in swine have given inconsistent results with regard to effects on the immune system. For example, maternal stress during gestation has been shown to either increase, decrease, or have no effect on the proliferative response of lymphocytes to mitogens in their offspring after birth in different models [42,43]. Also, the offspring of restraint-stressed dams challenged with LPS actually had lower cortisol levels and exhibited a heightened febrile response to LPS challenge [44]. It is clear that differences in the nature, timing, and duration of the developmental insult are very important in determining the outcome of programming events.

From a genetic standpoint, it is interesting to note that intensive selection for production traits such as body weight and lean growth in livestock species tends to have a negative impact on immunocompetence. For example, broiler chickens selected for a high birth weight exhibited lower antibody responses than control lines, and turkeys selected for high body weight exhibited higher mortality when challenged with a bacterial or viral pathogen [45,46]. Similar trends have also been observed in pigs [47]. It has been hypothesized that animals with high production trait potential may allocate metabolic resources primarily towards production related biological processes, such as muscle development, at the detriment of other processes such as immune system maintenance [48]. Ultimately this could result in an inadequate immune response to infection. Whether this negative correlation between production traits and immune traits could have contributed to the increased severity of disease in HBW pigs is unclear. Absolute differences in body weight were maintained from birth through to influenza challenge, although there was no difference in growth rates between birth weight groups [15]. Also, the transcriptional analysis suggested that the greater disease severity in HBW pigs was more likely attributable to an excessive, immunopathologic activation than a reduced immune capacity to fight infection.

Irrespective of litter birth weight phenotype, a range in severity of disease following influenza challenge was found, particularly in regard to lung pathology [15]. The inflammatory response was one of the most enriched functional annotation terms identified by GSEA for this comparison, together with associated pro-inflammatory cytokine pathways for IFN-γ, TNF-α, and IL-6 signaling. In the early stages of infection, inflammation plays an important role in limiting virus replication in the lung of infected pigs. However, an excessive inflammatory response is responsible for much of the pathology associated with influenza. DEGs identified as upregulated in SUS pigs include the pro-inflammatory cytokine genes *IL6* and *IL8*, and chemokine genes *CCL2* and *CXCL2*. All four genes are good candidates for driving disease pathology during influenza infection. Levels of both IL-6 and IL-8 are known to correlate with virus titers in lung of H3N2 influenza A-infected swine, and IL-6 levels also correlate with disease severity [1]. Elevated amounts of both cytokines in the lung have also been seen in severe cases of seasonal influenza infection in man [49], and IL-6 has been postulated as a biomarker for disease severity associated with pandemic H1N1 infection [50]. CCL2, a macrophage chemo-attractant, is also heavily involved in influenza pathogenesis. Mice that lack the gene for its receptor on the macrophage cell surface, CCR2, exhibit a significant reduction in macrophage migration to the lung and a lower rate of mortality upon influenza infection [51]. CCL2 has also recently been proposed as a biomarker for prediction of symptomatic influenza infection in humans [52]. Finally, antibodies against the CXCL2 protein, a neutrophil chemo-attractant, were found to reduce mortality from influenza-associated pneumonia in mice [53]. The cytokine expression profile of SUS pigs in this study is broadly similar to that of previous research into the host response to influenza virus at the gene expression level [54–56]. For example *IL1RN*, *IL6*, *CCL2*, and *CXCL10* were all associated with SUS pigs in this study and pigs infected with H1N2 influenza reported by Skovgaard *et al* [56]. Of the major cytokines involved in host response to influenza, only *IL8* expression was expressed differently between experiments (upregulated in SUS pigs, downregulated in H1N2 infected pigs). *CXCL10* mRNA was predicted to be the target of multiple microRNAs that are upregulated following infection, which could indicate that endogenous regulation of *CXCL10* expression is required for the resolution of infection without excessive tissue damage.

Other pathologic features of influenza infection in the lung were also represented in the gene expression profile of SUS pigs. Infiltration of the lung by neutrophils is part of the early innate immune response to influenza, and an important contributor to the overall pathology. The previously mentioned *IL8* and *CXCL2* both attract neutrophils to the infection site. Also *TCN1*, which encodes a major constituent of the secondary granules of neutrophils, is the most upregulated genes in the lung of SUS pigs. Another feature that can be associated with severe influenza infection in the lung is thrombosis [57]. Endothelial cell activation has been shown to upregulate cell surface adhesion molecules that favour platelet aggregation, and the genes for two such molecules, *SERPINE1* and *THBS1*, were more highly expressed in SUS pigs. Apoptosis of alveolar epithelial cells and leukocytes causes significant damage to the respiratory epithelium in severe cases of influenza-based pneumonia. *CCL2* also has a central role in this process by attracting CCR2+ macrophages from the blood. These cells express the tumour necrosis factor-related apoptosis inducing ligand (TRAIL) and induce apoptosis of airway epithelial cells [58]. Several other pro-apoptotic genes including *TNFRSF12A*, *DDIT3*, and *CDKN1A* were upregulated in SUS animals, indicating that they may also contribute to apoptotic events during influenza infection in swine.

Finally, a number of innate antiviral genes including *ISG15*, *OAS1*, *OAS2*, and *OASL* were upregulated in SUS pigs. In addition, GSEA identified interferon-associated gene sets and additional ISGs whose expression correlated with SUS phenotype, such as *MX1* and *IFI35*. Type I interferons inhibit protein synthesis in infected and neighbouring cells, which probably explains why ribosomal gene expression is greater in RES than SUS pigs. Many of the ISGs identified have antiviral functions, but the magnitude of their expression is positively associated with increased disease severity in this study rather than disease resilience. It may be that their elevated expression in SUS compared to RES pigs was in response to increased viral load in the lung of these animals at the 48-hour time-point. Clearly, if these and other antiviral genes do contribute to a disease resistance phenotype in swine influenza then it must be during the earlier stages of infection before the virus is well established in the lung tissue, and before it is possible to categorize animals into different susceptibility groups.

## Conclusions

In this study, the effect of a litter birth weight phenotype on the transcriptomic and epigenetic responses in the lung following experimental influenza infection in swine was investigated. The transcriptomic response of immune genes to infection was lower in the LBW than HBW group of pigs, consistent with the lower disease burden previously reported in the low birth weight group. This was contrary to our original hypothesis that the low birth weight group would be more susceptible to influenza due to the detrimental effects of ‘prenatal programming’ on the immune system. Furthermore, no evidence was found for epigenetic changes in the promoter regions of genes that were differentially expressed between LBW and HBW groups. A second analysis using a subset of animals classified into divergent susceptibility groups on the basis of lung pathology found that susceptible pigs mounted a more robust inflammatory response to infection that was likely driven by a group of pro-inflammatory cytokine genes that include *IL6* and *CCL2*.

## Supporting Information

S1 Appendix RT-qPCR Assay Information. (DOCX)

S2 Appendix Lists of Differentially Expressed Genes. (XLSX)

S3 Appendix GSEA Results. (XLSX)
