# Supplementary material for: Transcriptomic and Epigenetic Profiling of the Lung of Influenza-Infected Pigs: A Comparison of Different Birth Weight and Susceptibility Groups
Source: PLoS One. 2015 Sep 22;10(9):e0138653. doi: 10.1371/journal.pone.0138653 (PMC4578952; doi:10.1371/journal.pone.0138653)

**Supplementary Figure 1: MIQE Information for RT-qPCR Assays**

18S ribosomal RNA

Gene symbol: 18S rRNA

Sequence accession ID: NR_002170

Forward primer: GACAAATCGCTCCACCAACT

Reverse primer: CCTGGGGCTTAATTTGACTC

Amplicon length: 134 bp

Forward primer location: Within the only exon of the gene

Reverse primer location: Within the only exon of the gene

Calibration curve:

Efficiency (from calibration curve): 1.09

R^2^: 0.9983

Slope: -2.161

Cq of NTC: 31.86

Cq of no reverse transcriptase control: 31.95

Cq Mean: 13.28

Cq Standard Deviation: 0.46


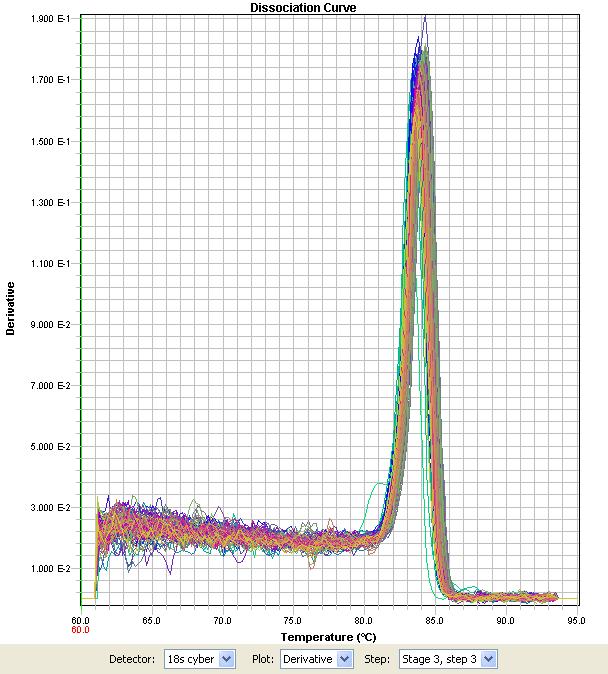


Apolipoprotein A1

Gene Symbol: APOA1

Sequence ID: ENSSSCT00000034599

Forward Primer: AAAACACCTCAACCTGAAACTCCTG

Reverse Primer: AGCTGCTCACGCACTTTGG

Forward Primer Location: Spans boundary of exons 3 and 4

Reverse Primer Location: Within exon 4

Amplicon Size: 74 bp

Calibration curve:

Efficiency (from calibration curve): 0.91

R^2^: 0.9992

Slope: -2.482

Cq of NTC: No amplification detected

Cq of no reverse transcriptase control: No amplification detected

Cq Mean: 29.71

Cq Standard deviation: 0.79

Dissociation curve:


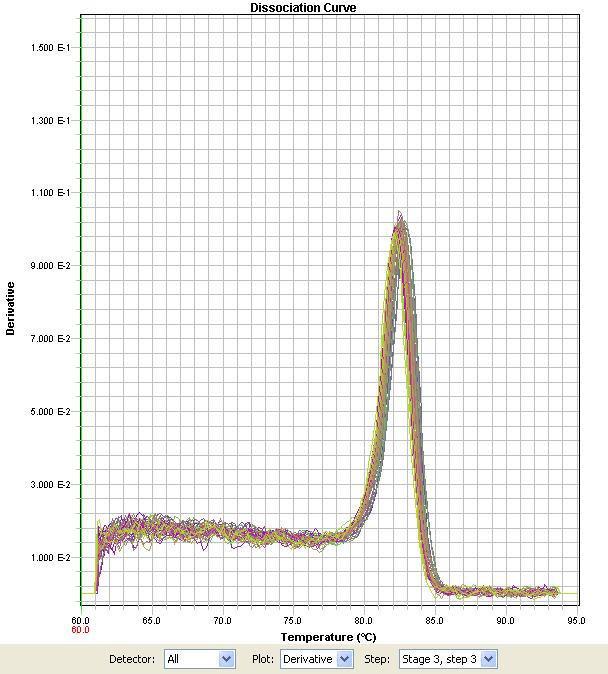


Complement component 1, r subcomponent

Gene Symbol: C1R

Sequence ID: ENSSSCT00000000733

Forward Primer: CAAGCTGTGGACCTGGATGAATG

Reverse Primer: AGCAGAAATAGCCGCCGATG

Forward Primer Location: Spans boundary of exons 3 and 4

Reverse Primer Location: Within exon 4

Amplicon Size: 111 bp

Calibration curve:

Efficiency (from calibration curve): 0.95

R^2^: 0.9969

Slope: -2.406

Cq of NTC: No amplification detected

Cq of no reverse transcriptase control: No amplification detected

Cq Mean: 23.35

Cq Standard deviation: 1.57

Dissociation curve:


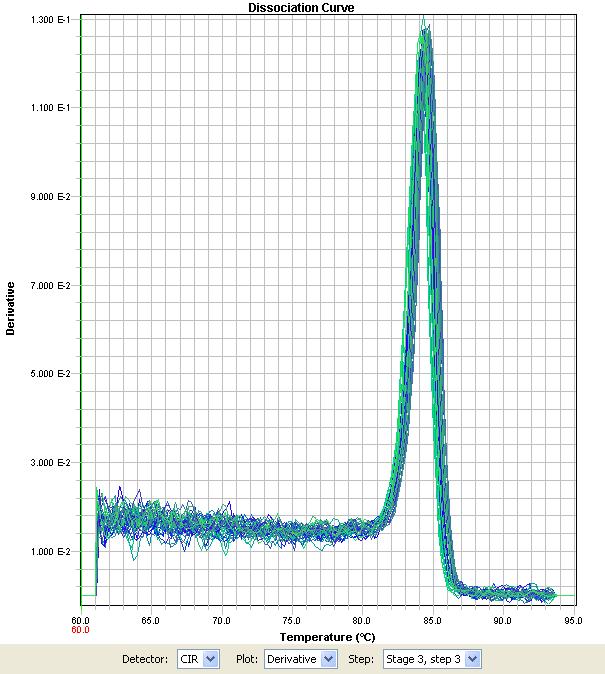


Complement component 1, s subcomponent

Gene Symbol: C1S

Sequence ID: NM001005349.1

Forward Primer: CGGATGGAAGTGAGGAGTATCG

Reverse Primer: TAAAGGGCTCGCTGGGGATG

Forward Primer Location: Spans boundary of putative exons 10 and 11

Reverse Primer Location: Within exon 11

Amplicon Size: 113 bp

Calibration curve:

Efficiency (from calibration curve): 0.93

R^2^: 0.9988

Slope: -2.444

Cq of NTC: No amplification detected

Cq of no reverse transcriptase control: No amplification detected

Cq Mean: 22.74

Cq Standard deviation: 1.39

Dissociation curve:


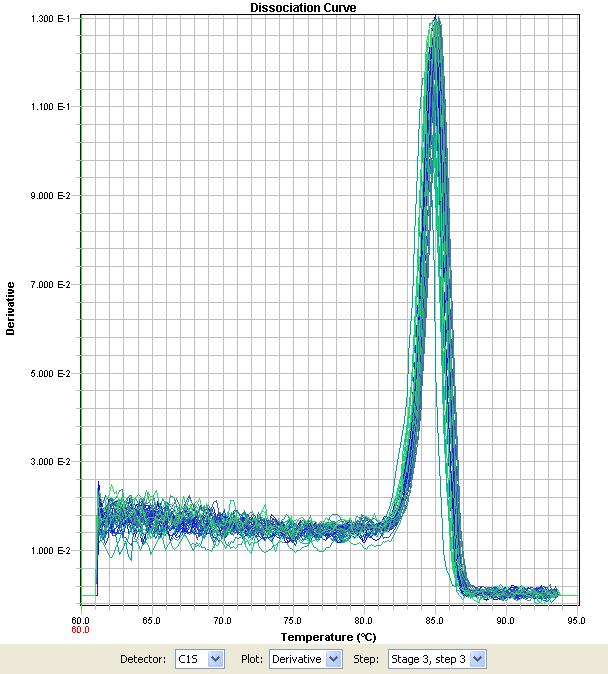


Chemokine (C-C motif) Ligand 17

Gene Symbol: CCL17

Sequence ID: ENSSSCT00000023793

Forward Primer: ACGCCATTGTGCTTGTGACC

Reverse Primer: CGAACGGTCCAAGGAGAAATC

Forward Primer Location: Spans boundary of exons 2 and 3

Reverse Primer Location: Within exon 3

Amplicon Size: 149 bp

Calibration curve:

Efficiency (from calibration curve): 1.03

R^2^: 0.9973

Slope: -2.273

Cq of NTC: 36.49

Cq of no reverse transcriptase control: 36.03

Cq Mean: 30.30

Cq standard deviation: 1.45

Dissociation curve:


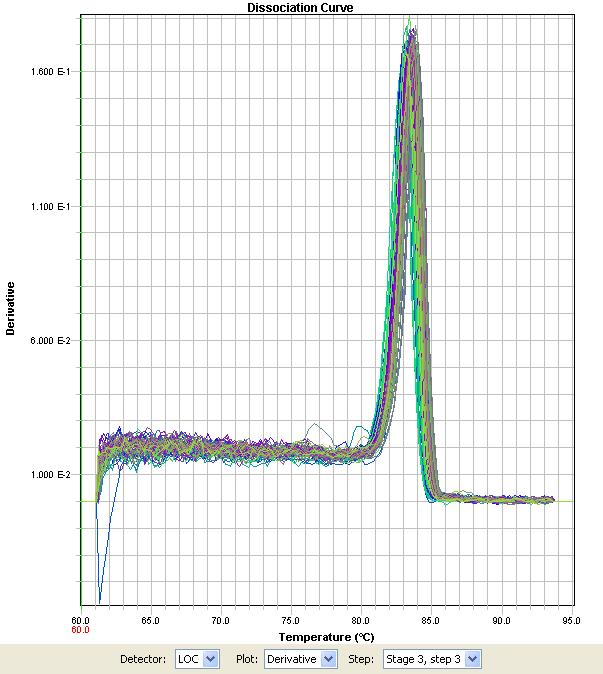


Chemokine (C-C motif) Ligand 2

Gene Symbol: CCL2

Sequence ID: ENSSSCT00000019290

Forward Primer: CCTTGCCCAGCCAGATGC

Reverse Primer: AGATCACTGCTTCTTTAGGACACTTGC

Forward Primer Location: Spans boundary of exons 2 and 3

Reverse Primer Location: Within exon 3

Amplicon Size: 133 bp

Calibration curve:

Efficiency (from calibration curve): 0.94

R^2^: 0.9991

Slope: -2.424

Cq of NTC: No amplification detected

Cq of no reverse transcriptase control: No amplification detected

Cq Mean: 23.82

Cq Standard deviation: 2.22

Dissociation curve:


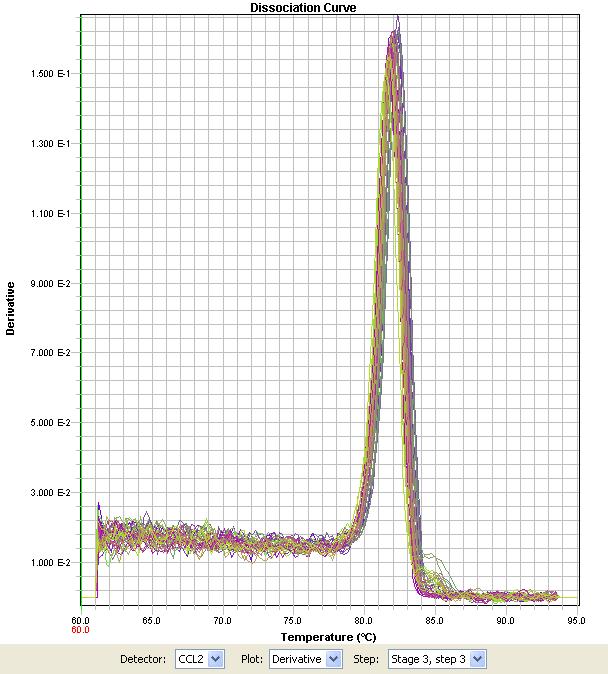


Chemokine (C-C motif) Ligand 25

Gene Symbol: CCL25

Sequence ID: ENSSSCT00000014845

Forward Primer: ACAGCTATTCCAGGACCATGAGC

Reverse Primer: AGCCTCAGGTGCCAGGATTG

Forward Primer Location: Spans boundary of exons 5 and 6

Reverse Primer Location: Within exon 6

Amplicon Size: 152 bp

Calibration curve:

Efficiency (from calibration curve): 1.02

R^2^: 0.9969

Slope: -2.293

Cq of NTC: 37.52

Cq of no reverse transcriptase control: 37.44

Mean Cq: 30.60

Cq Standard deviation: 1.13

Dissociation curve:


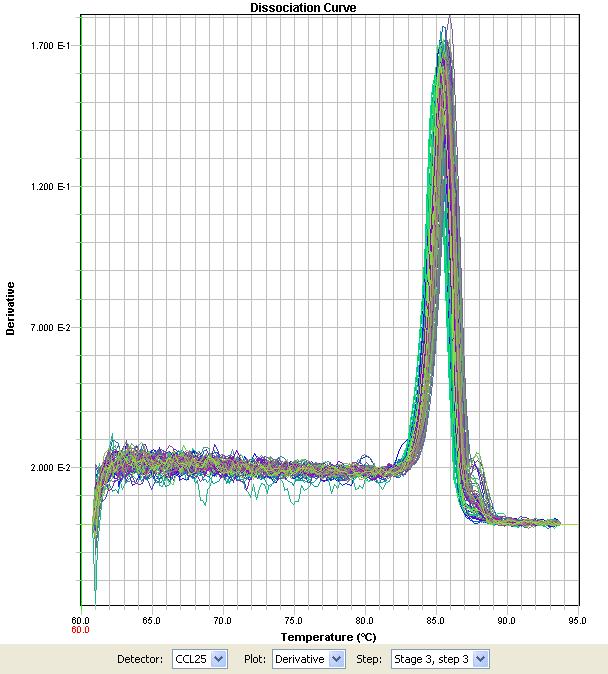


Chemokine (C-C motif) Ligand 28

Gene Symbol: CCL28

Sequence ID: ENSSSCT00000018375

Forward Primer: GGCTGCTGTCATCCTTCATGTC

Reverse Primer: GGCTGCTTGTTCTTTCATCCAC

Forward Primer Location: Spans boundary of exons 2 and 3

Reverse Primer Location: Within exon 3

Amplicon Size: 90 bp

Calibration curve:

Efficiency (from calibration curve): 1.05

R^2^: 0.9923

Slope: -2.248

Cq of NTC: No amplification detected

Cq of no reverse transcriptase control: No amplification detected

Cq Mean: 27.09

Cq Standard deviation: 1.17

Dissociation curve:


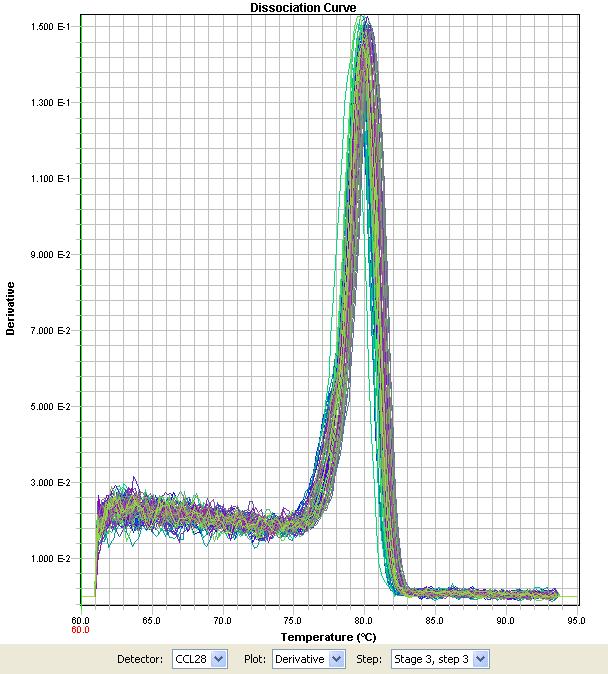


CD 1 Antigen (CD1.1)

Gene Symbol: PCD1A

Sequence ID: ENSSSCT00000022296

Forward Primer: TCCAGCGACAAGTAAGACCTGAG

Reverse Primer: GCCAGAGACGTGACAAACCAG

Forward Primer Location: Spans boundary of exons 3 and 4

Reverse Primer Location: Within exon 4

Amplicon Size: 88 bp

Calibration curve:

Efficiency (from calibration curve): 1.00

R^2^: 0.9954

Slope: -2.324

Cq of NTC: No amplification detected

Cq of no reverse transcriptase control: No amplification detected

Mean Cq: 27.60

Cq Standard deviation: 1.12

Dissociation curve:


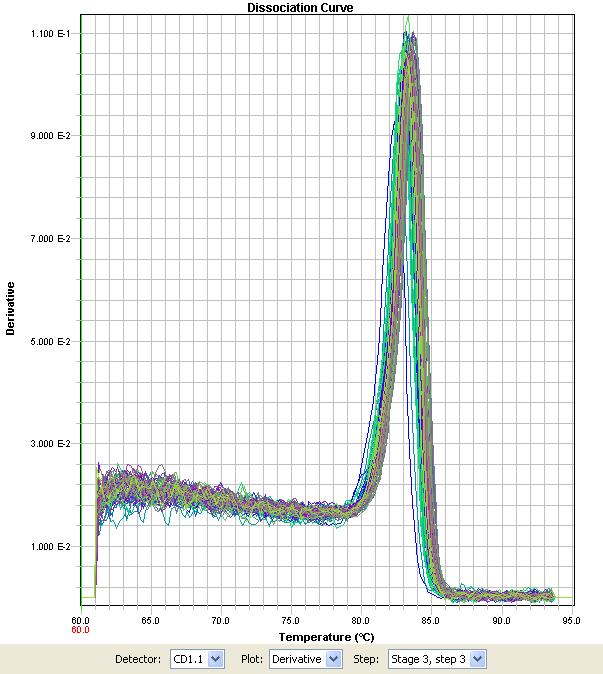


Ceruloplasmin

Gene Symbol: CP

Sequence ID: ENSSSCT00000033091

Forward Primer: CCAATACCAGCACAGGGGCATTTATAC

Reverse Primer: GCAGTGGAGTAACCAGGTTCCAG

Forward Primer Location: Spans boundary of exons 17 and 18

Reverse Primer Location: Within exon 18

Amplicon Size: 111 bp

Calibration curve:

Efficiency (from calibration curve): 0.90

R^2^: 0.9935

Slope: -2.515

Cq of NTC: No amplification detected

Cq of no reverse transcriptase control: No amplification detected

Cq Mean: 28.83

Cq Standard deviation: 1.37

Dissociation curve:


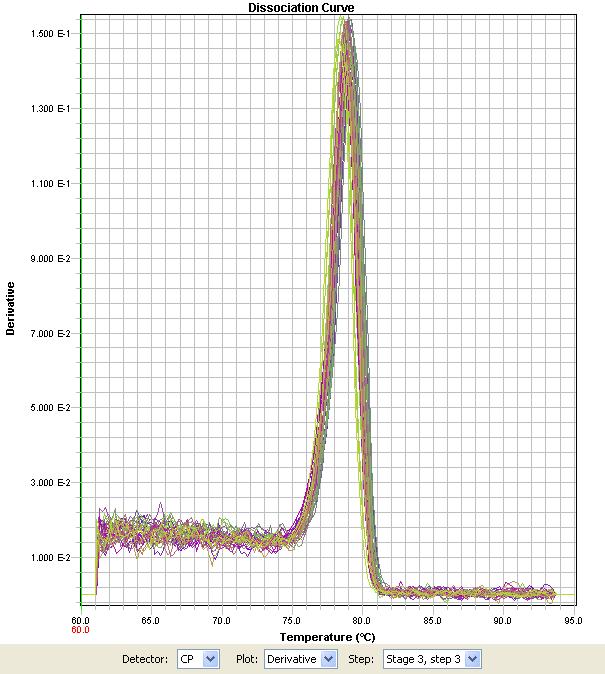


Cellular Retinoic Acid Binding Protein 1

Gene Symbol: CRABP1

Sequence ID: ENSSSCT00000025681

Forward Primer: AAGGCACTGGGTGTGAACG

Reverse Primer: CCGTGGTGGATGTCTTGATG

Forward Primer Location: Spans boundary of exons 1 and 2

Reverse Primer Location: Within exon 2

Amplicon Size: 114 bp

Calibration curve:

Efficiency (from calibration curve): 0.98

R^2^: 0.9938

Slope: -2.363

Cq of NTC: No amplification detected

Cq of no reverse transcriptase control: No amplification detected

Cq Mean: 26.97

Cq Standard deviation: 1.11

Dissociation curve:


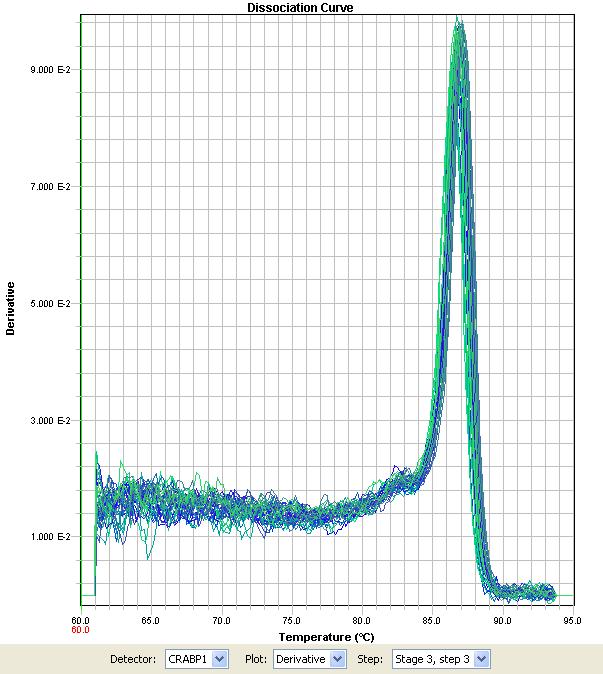


Chemokine (C-X-C motif) Ligand 2

Gene Symbol: CXCL2

Sequence ID: ENSSSCT00000034640

Forward Primer: GATGCTAAACAAGAGCAGTGCCAAC

Reverse Primer: CCCCAGGGGCTATTTGCTTC

Forward Primer Location: Spans boundary of exons 3 and 4

Reverse Primer Location: Within exon 4

Amplicon Size: 113 bp

Calibration curve:

Efficiency (from calibration curve): 0.92

R^2^: 0.9975

Slope: -2.465

Cq of NTC: 38.62

Cq of no reverse transcriptase control: No amplification detected

Cq Mean: 28.23

Cq Standard deviation: 1.47

Dissociation curve:


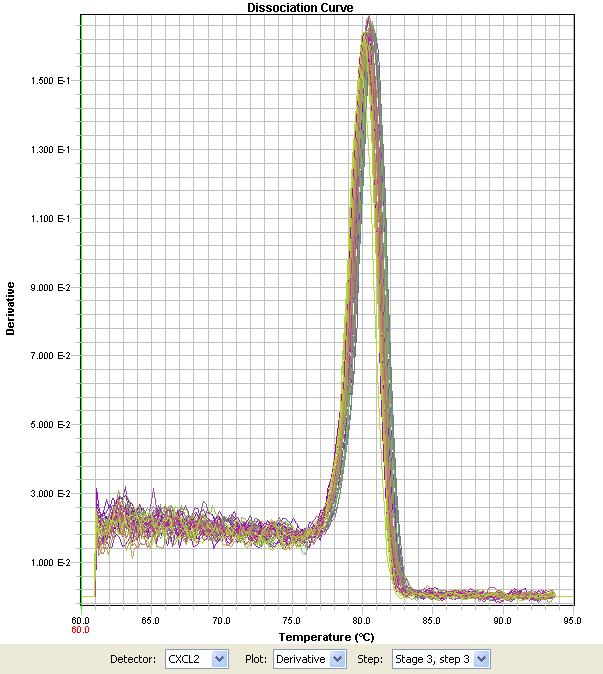


Fc Fragment of IgE, high affinity 1, receptor for, alpha polypeptide

Gene Symbol: FCER1A

Sequence ID: ENSSSCT00000007026

Forward Primer: ACCATACAAACAGATTCCCCTTCAG

Reverse Primer: TGCTGGGTTGAGATCAGTAGCC

Forward Primer Location: Spans boundary of exons 4 and 5

Reverse Primer Location: Within exon 5

Amplicon Size: 118 bp

Calibration curve:

Efficiency (from calibration curve): 0.90

R^2^: 0.989

Slope: -2.505

Cq of NTC: No amplification detected

Cq of no reverse transcriptase control: No amplification detected

Cq Mean: 28.52

Cq Standard deviation: 1.44

Dissociation curve:


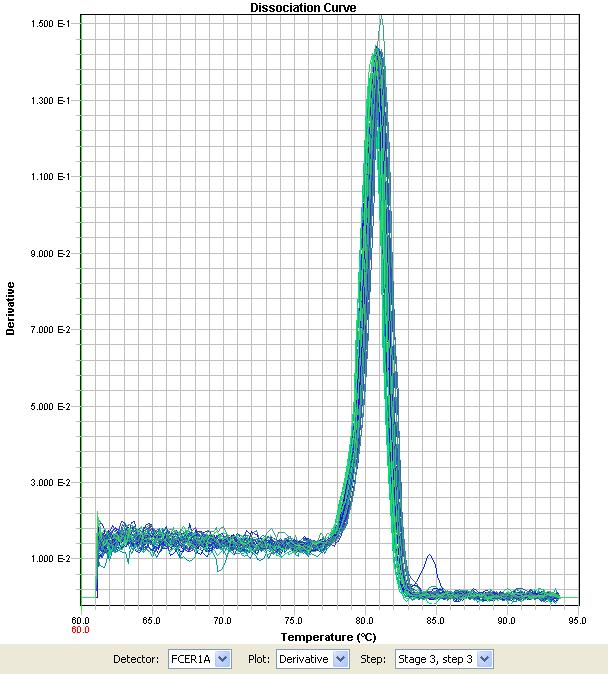


Growth Differentiation Factor 15

Gene Symbol: GDF15

Sequence ID: NM_001174056.1

Forward Primer: CGTGCCCGAGCCACTTCC

Reverse Primer: GCCATCGCTGTCCTGGTG

Forward Primer Location: Within putative exon 2

Reverse Primer Location: Within putative exon 2

Amplicon Size: 128 bp

Calibration curve:

Efficiency (from calibration curve): 0.99

R^2^: 0.9752

Slope: -2.331

Cq of NTC: 38.30

Cq of no reverse transcriptase control: 38.69

Cq Mean: 31.46

Cq Standard deviation: 1.11

Dissociation curve:


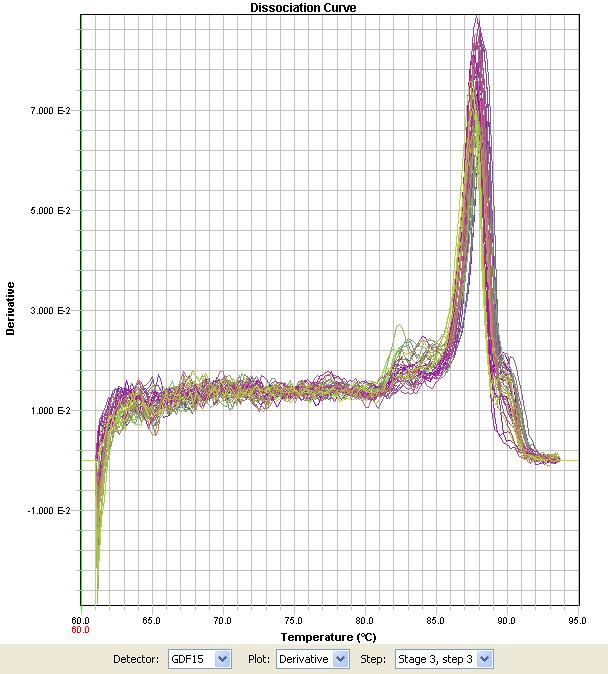


Interferon alpha

Gene Symbol: IFNA

Sequence ID: NM_214393.1

Forward Primer: CCCCAACCTCAGCCTTCCTC

Reverse Primer: AGGCAGGTCACAGCCCAGAG

Forward Primer Location: Within only exon

Reverse Primer Location: Within only exon

Amplicon Size: 77 bp

Calibration curve:

Efficiency (from calibration curve): 1.00

R^2^: 0.9716

Slope: -2.321

Cq of NTC: No amplification detected

Cq of no reverse transcriptase control: No amplification detected

Cq Mean: 27.91

Cq Standard deviation: 0.74

Dissociation curve:


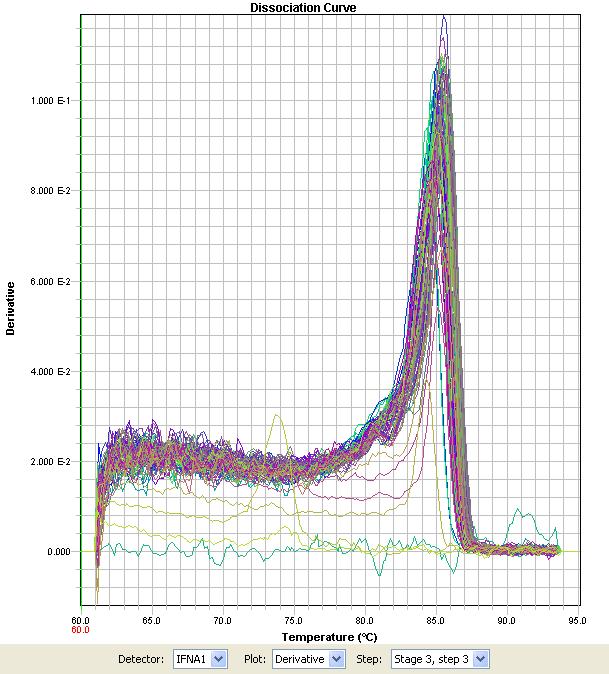


Interferon gamma

Gene symbol: IFNG

Sequence ID: NM_213948

Forward primer: TTTGAAGAATTGGAAAGAGGAGAGTGA

Reverse primer: GCTCCTTTGAATGGCCTGGTT

Amplicon length: 112 bp

Forward primer location: Spans boundary of exons 2 and 3.

Reverse primer location: Within exon 3

Calibration curve:

Efficiency (from calibration curve): 0.92

R^2^: 0.989

Slope: -2.470

Cq of NTC: No amplification detected

Cq of no reverse transcriptase control: No amplification detected

Cq Mean: 34.29

Cq Standard deviation: 1.51

Dissociation curve:


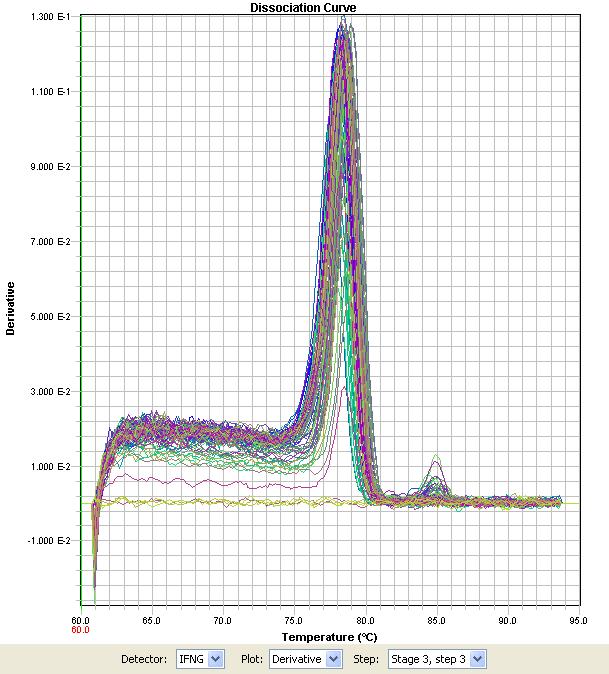


Interleukin 1 beta

Gene symbol: IL1B

Sequence ID: NM_214055.1

Forward primer: TGATGGCTAACTACGGTGACAACA

Reverse primer: CTCAGAGAACCAAGGTCCAGGTTT

Amplicon length: 103 bp

Forward primer location: Spans boundary of exons 2 and 3.

Reverse primer location: Within exon 4

Calibration curve:

Efficiency (from calibration curve): 1.05

R^2^: 0.9672

Slope: -2.253

Cq of NTC: No amplification detected

Cq of no reverse transcriptase control: No amplification detected

Cq Mean: 28.55

Cq Standard deviation: 1.22

Dissociation curve:


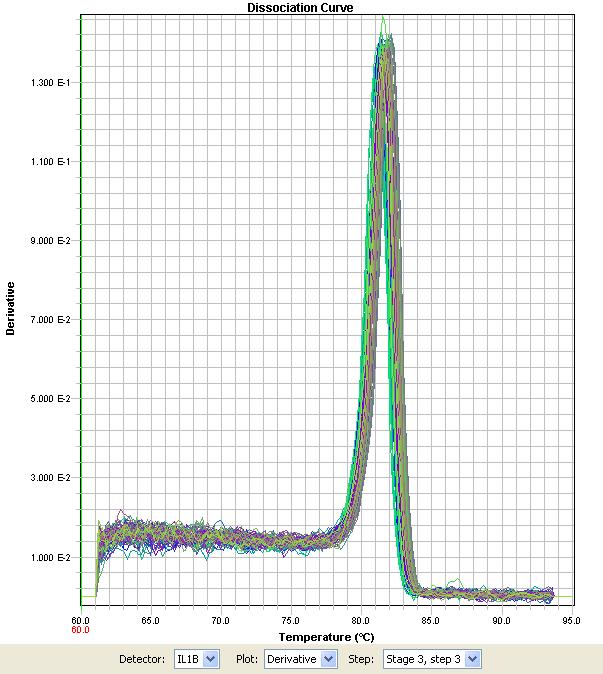


Interleukin 1 receptor antagonist

Gene symbol: IL1RN

Sequence ID: AK346235.1

Forward primer: CAAGCCTTCAGAATCTGGGATGTC

Reverse primer: GGCTCAACAGGCACCACATC

Amplicon length: 127 bp

Forward primer location: Spans boundary of exons 1 and 2.

Reverse primer location: Within exon 3

Calibration curve:

Efficiency (from calibration curve): 1.03

R^2^: 0.9994

Slope: -2.282

Cq of NTC: 37.92

Cq of no reverse transcriptase control: 37.11

Cq Mean: 22.61

Cq Standard deviation: 1.55

Dissociation curve:


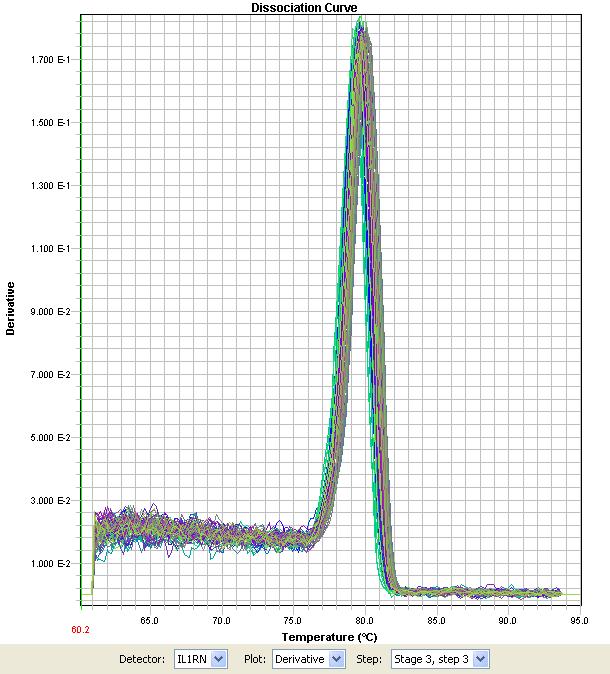


Interleukin 6

Gene symbol: IL6

Sequence ID: ENSSSCT00000025647

Forward primer: CTCTCCACAAGCGCCTTTAGTC

Reverse primer: GCATTTTGTCTGAGGTGGCATC

Amplicon length: 126 bp

Forward primer location: Spans boundary of exons 1and 2.

Reverse primer location: Within exon 2

Calibration curve:

Efficiency (from calibration curve): 1.01

R^2^: 0.9812

Slope: -2.300

Cq of NTC: 38.23

Cq of no reverse transcriptase control: 38.46

Cq Mean: 29.69

Cq Standard deviation: 1.33

Dissociation curve:


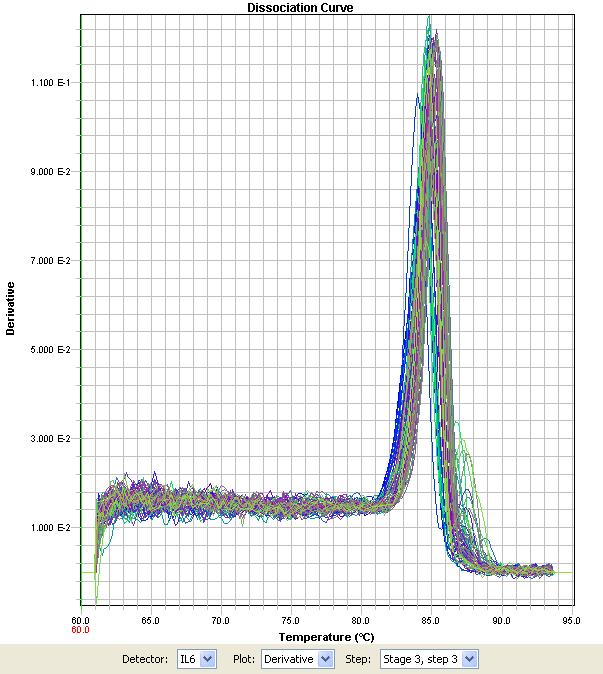


Interleukin 8

Gene symbol: IL8

Sequence ID: NM_213867.1

Forward primer: TGAAGAGAACTGAGAAGCAACAACAAC

Reverse primer: TCTGGCAACCCTATGTCTGACC

Amplicon length: 139 bp

Forward primer location: Spans boundary of exons 3 and 4.

Reverse primer location: Within exon 4

Calibration curve:

Efficiency (from calibration curve): 0.91

R^2^: 0.9565

Slope: -2.506

Cq of NTC: No amplification detected

Cq of no reverse transcriptase control: No amplification detected

Cq Mean: 26.17

Cq Standard deviation: 1.15

Dissociation curve:


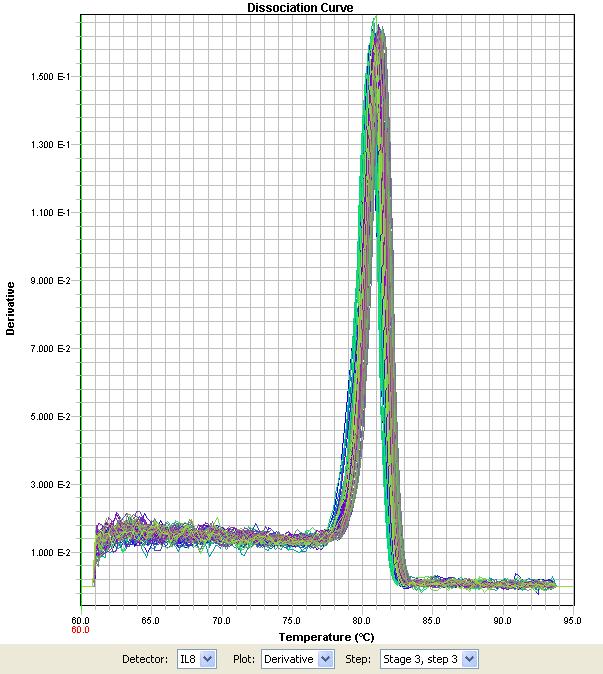


Interferon-stimulated gene 15

Gene symbol: ISG15

Sequence ID: ENSSSCT00000029764

Forward primer: CTGGAGGGTGGGGAGGGTAG

Reverse primer: GTCCCAGGGCCACCACATAG

Amplicon length: 84 bp

Forward primer location: Within only exon

Reverse primer location: Within only exon

Calibration curve:

Efficiency (from calibration curve): 0.90

R^2^: 0.9983

Slope: -2.501

Cq of NTC: No amplification detected

Cq of no reverse transcriptase control: No amplification detected

Cq Mean: 29.30

Cq Standard deviation: 0.89

Dissociation curve:


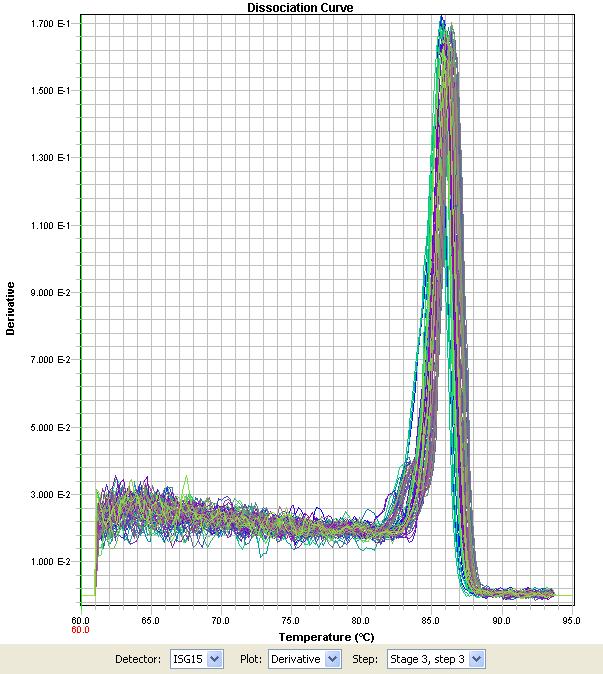


Influenza virus NS1A binding protein

Gene symbol: IVNS1ABP

Sequence ID: ENSSSCT00000016961

Forward primer: TCGGAAAAGACCTCAAATAACACTTAC

Reverse primer: GAGTTTTGGAGTGCTTGTTGGTG

Amplicon length: 119 bp

Forward primer location: Spans boundary of exons 4 and 5

Reverse primer location: Within exon 5

Calibration curve:

Efficiency (from calibration curve): 1.00

R^2^: 0.9853

Slope: -2.320

Cq of NTC: 33.98

Cq of no reverse transcriptase control: 34.71

Cq Mean: 27.36

Cq Standard deviation: 1.03

Dissociation curve:


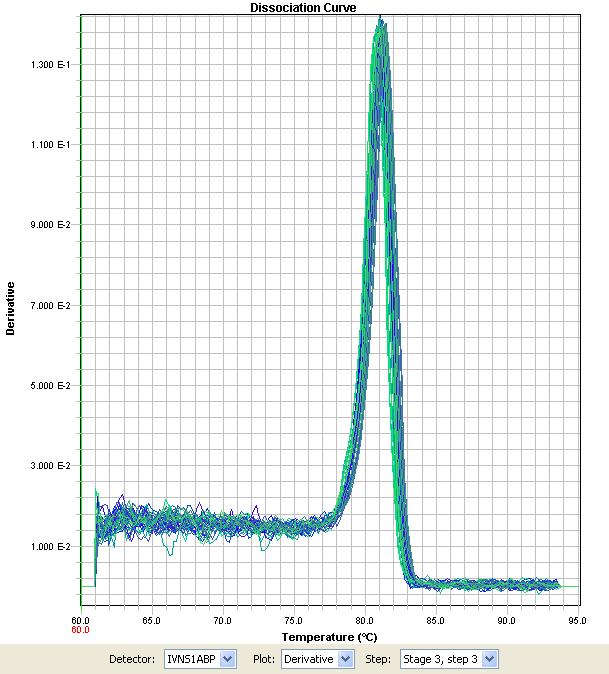


Lymphoid enhancer-binding factor 1

Gene symbol: LEF1

Sequence ID: ENSSSCT00000010018

Forward primer: GGAATCTACATCAGGTACAGGTCCAAG

Reverse primer: GGGCCAGGTTTTCAACAAGC

Amplicon length: 145 bp

Forward primer location: Spans boundary of exons 10 and 11

Reverse primer location: Within exon 12

Calibration curve:

Efficiency (from calibration curve): 0.94

R^2^: 0.9869

Slope: -2.437

Cq of NTC: No amplification detected

Cq of no reverse transcriptase control: No amplification detected

Cq Mean: 24.62

Cq Standard deviation: 0.81

Dissociation curve:


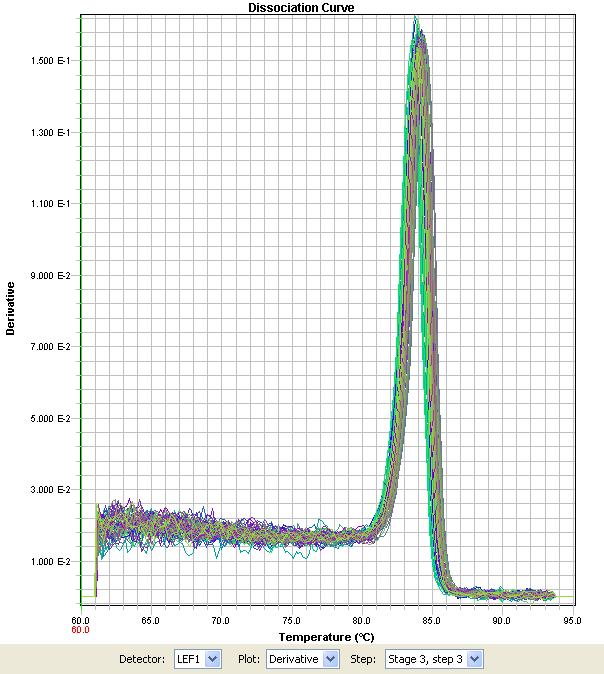


Mesenchyme homeobox 1

Gene symbol: MEOX1

Sequence ID: ENST00000318579

Forward primer: AGGAGAGTTCAGACAATCAGGAGAAC

Reverse primer: CAGGTAGTTGTGATGAGCAAATTCAG

Amplicon length: 131 bp

Forward primer location: Spans boundary of exons 1 and 2

Reverse primer location: Within exon 2

Calibration curve:

Efficiency (from calibration curve): 0.93

R^2^: 0.9961

Slope: -2.445

Cq of NTC: No amplification detected

Cq of no reverse transcriptase control: No amplification detected

Cq Mean: 34.12

Cq Standard deviation: 1.31

Dissociation curve:


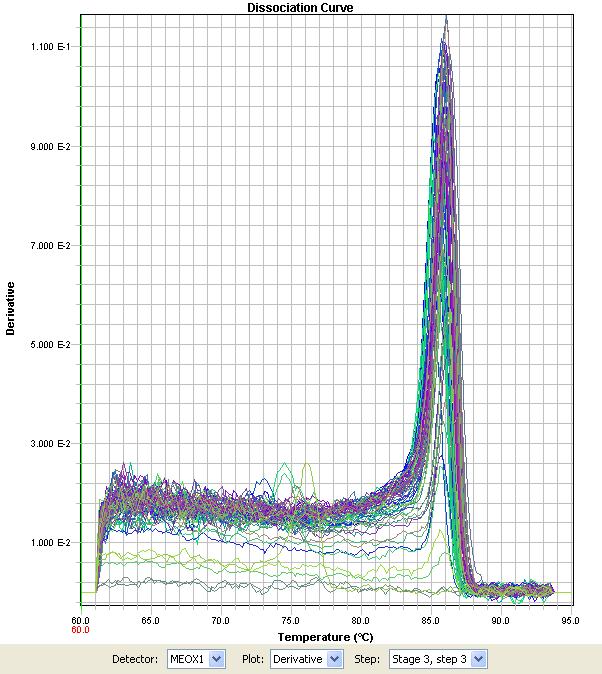


NADH-ubiquinone oxidoreductase chain 4

Gene symbol: MT-ND4

Sequence ID: ENSSSCT00000019682

Forward primer: AAACCAAACAGAACGACTCAATGC

Reverse primer: GAAGTTTAGTGAGCCTGTGGTATTTTG

Amplicon length: 117 bp

Forward primer location: Within only exon

Reverse primer location: Within only exon

Calibration curve:

Efficiency (from calibration curve): 1.00

R^2^: 0.9854

Slope: -2.317

Cq of NTC: No amplification detected

Cq of no reverse transcriptase control: No amplification detected

Cq Mean: 22.72

Cq Standard deviation: 0.95

Dissociation curve:


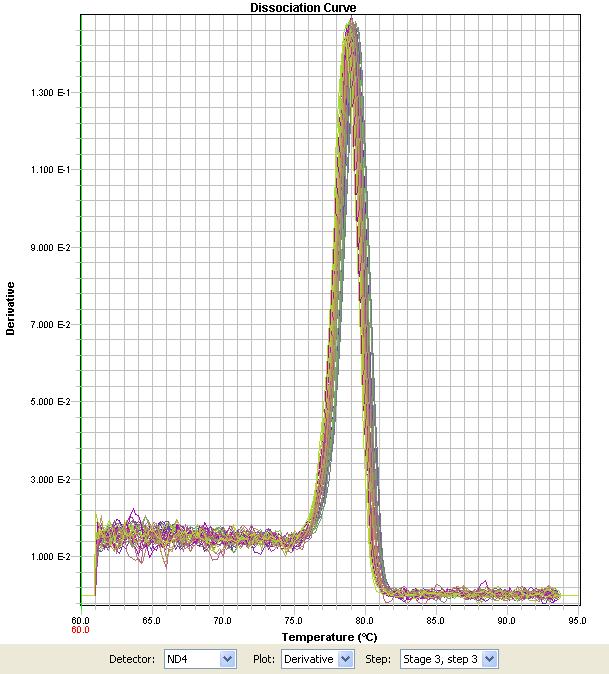


NADH-ubiquinone oxidoreductase chain 5

Gene symbol: MT-ND5

Sequence ID: ENSSSCT00000019686

Forward primer: CTACACAAACGCCTGAGCCCTAC

Reverse primer: GAATGCAAAGAAGATAATTCGAGTGC

Amplicon length: 87 bp

Forward primer location: Within only exon

Reverse primer location: Within only exon

Calibration curve:

Efficiency (from calibration curve): 1.06

R^2^: 0.9902

Slope: -2.228

Cq of NTC: 37.42

Cq of no reverse transcriptase control: 36.67

Cq Mean: 22.85

Cq standard deviation: 0.98

Dissociation curve:


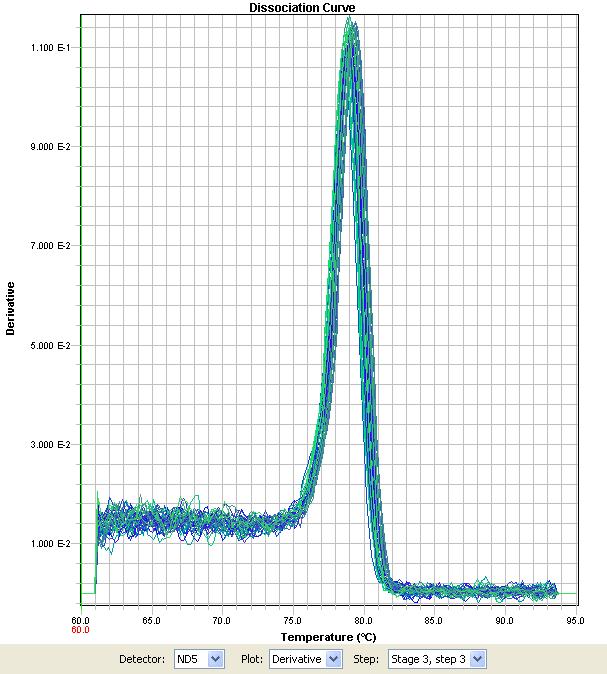


2,-5,-oligoadenylate synthetase 1

Gene symbol: OAS1

Sequence ID: ENSSSCT00000010832

Forward primer: GATGCCCTGGGTCAGTGGAC

Reverse primer: TCCGTGAAGCAGGTGGAAAAC

Amplicon length: 115 bp

Forward primer location: Spans boundary of exons 2 and 3

Reverse primer location: Within exon 3

Calibration curve:

Efficiency (from calibration curve): 1.10

R^2^: 0.9882

Slope: -2.170

Cq of NTC: No amplification detected

Cq of no reverse transcriptase control: No amplification detected

Cq Mean: 33.28

Cq Standard deviation: 1.43

Dissociation curve:


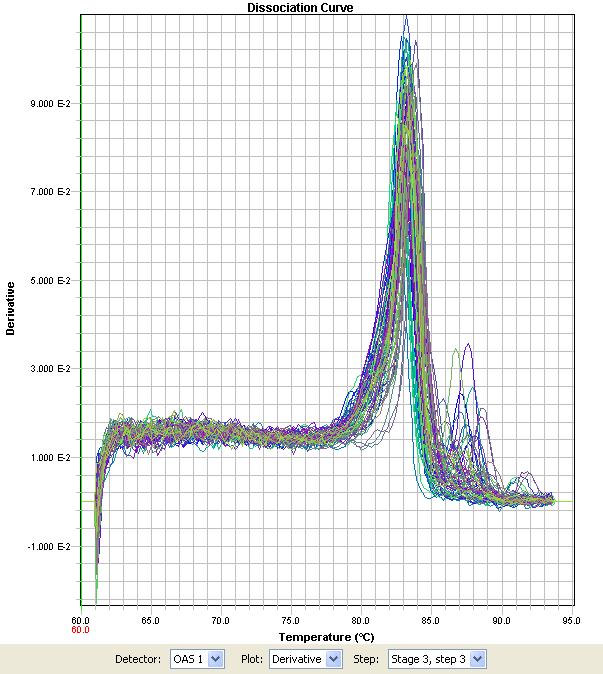


2,-5,-oligoadenylate synthetase 2

Gene symbol: OAS2

Sequence ID: ENSSSCT00000010831

Forward primer: CATCTTGGAAATATCTACCTGCATCAC

Reverse primer: GATGTCCACGGCCTTCTTGG

Amplicon length: 122 bp

Forward primer location: Spans boundary of exons 3,4, and 5

Reverse primer location: Within exon 5

Calibration curve:

Efficiency (from calibration curve): 0.98

R^2^: 0.9989

Slope: -2.350

Cq of NTC: No amplification detected

Cq of no reverse transcriptase control: 38.96

Cq Mean: 24.82

Cq Standard deviation: 1.34

Dissociation curve:


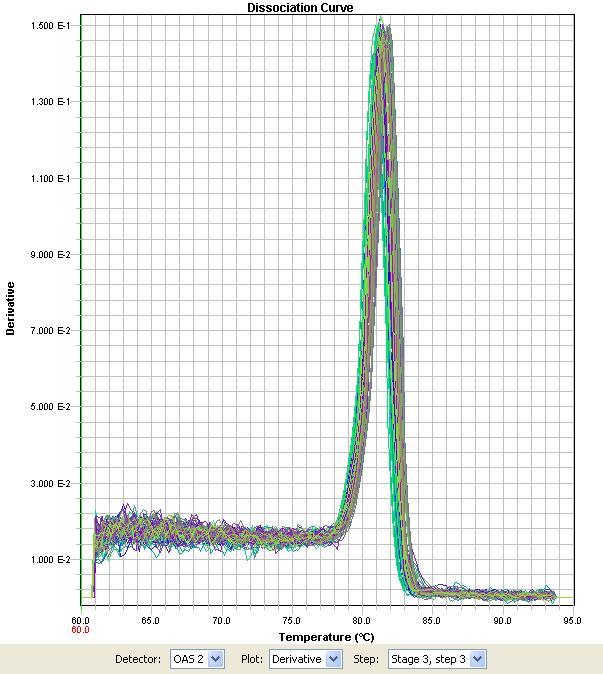


2,-5,-oligoadenylate synthetase like

Gene symbol: OASL

Sequence ID: ENSSSCT00000010872

Forward primer: AGGTGCTAGAGGTGGGCTCCTTC

Reverse primer: CGGAAGCTGCGGAAACAG

Amplicon length: 96 bp

Forward primer location: Spans boundary of exons 1 and 2

Reverse primer location: Within exon 2

Calibration curve:

Efficiency (from calibration curve): 1.06

R^2^: 0.9902

Slope: -2.223

Cq of NTC: 37.33

Cq of no reverse transcriptase control: 37.36

Cq Mean: 31.26

Cq Standard deviation: 2.01

Dissociation curve:


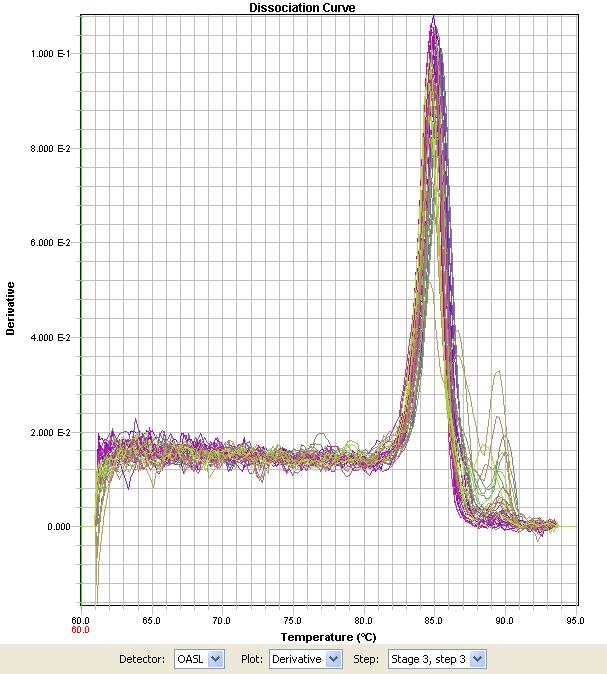


Serpin peptidase inhibitor clade E member 1

Gene symbol: SERPINE1

Sequence ID: ENSSSCT00000026956

Forward primer: CCTCCTCTACGGCCATTATCGTC

Reverse primer: ACCGTTCCTGTGGGGTTGTG

Amplicon length: 102 bp

Forward primer location: Spans boundary of exons 9 and 10

Reverse primer location: Within exon 11

Calibration curve:

Efficiency (from calibration curve): 0.87

R^2^: 0.9978

Slope: -2.579

Cq of NTC: No amplification detected

Cq of no reverse transcriptase control: No amplification detected

Cq Mean: 29.95

Cq Standard deviation: 1.41

Dissociation curve:


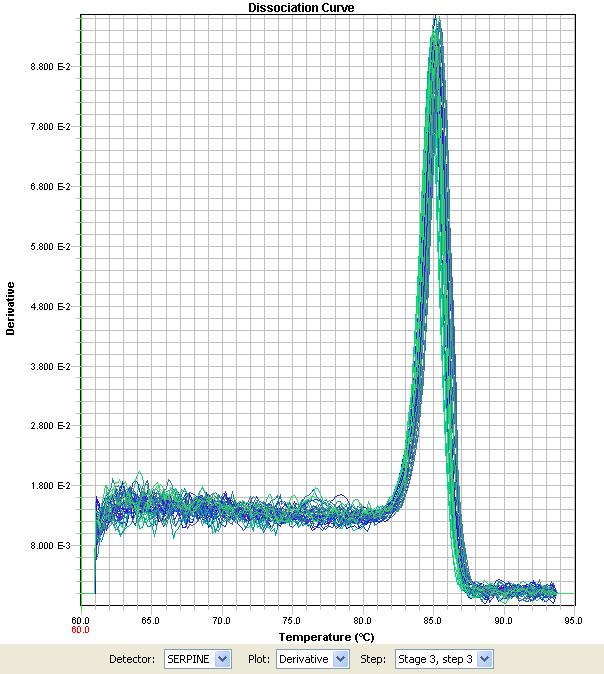


Serpin peptidase inhibitor clade G member 1

Gene symbol: SERPING1

Sequence ID: ENSSSCT00000034157

Forward primer: TCTACCTGAGTGCCAAGTGGAAGG

Reverse primer: GGGCCACAGGGTACTTCTTGC

Amplicon length: 122 bp

Forward primer location: Spans boundary of exons 6 and 7

Reverse primer location: Within exon 7

Calibration curve:

Efficiency (from calibration curve): 1.07

R^2^: 0.9939

Slope: -2.212

Cq of NTC: 39.44

Cq of no reverse transcriptase control: 38.21

Cq Mean: 23.85

Cq Standard deviation: 0.99

Dissociation curve:


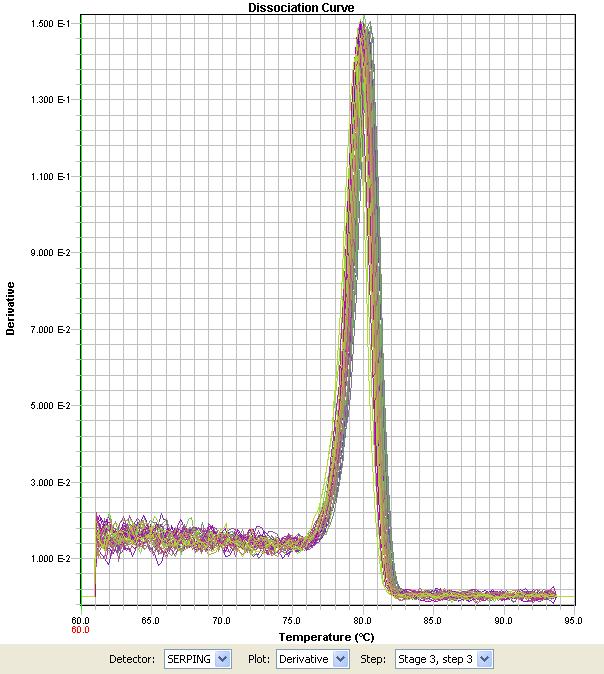


Stratifin

Gene symbol: SFN

Sequence ID: ENSSSCT00000003956

Forward primer: GAATGTGGTGGGTGGTCAGC

Reverse primer: TGAGATGGGTGTTCAGCAAGC

Amplicon length: 175 bp

Forward primer location: Within only exon

Reverse primer location: Within only exon

Calibration curve:

Efficiency (from calibration curve): 1.10

R^2^: 0.9943

Slope: -2.162

Cq of NTC: 37.88

Cq of no reverse transcriptase control: 38.70

Cq Mean: 30.18

Cq Standard deviation: 1.41

Dissociation curve:


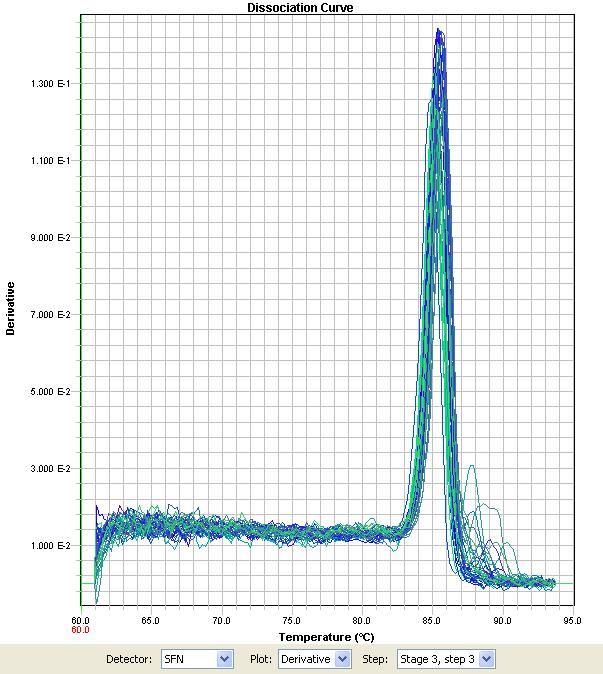


Sus scrofa MHC class II histocompatibility antigen SLA-DQA1

Gene symbol: SLA-DQA1

Sequence ID: ENSSSCT00000001616

Forward primer: GACTCTCCACAGGATTTCGTGTACC

Reverse primer: CTCCCCCACGTCGCTGTC

Amplicon length: 94 bp

Forward primer location: Spans boundary of exons 1 and 2

Reverse primer location: Within exon 2

Calibration curve:

Efficiency (from calibration curve): 1.10

R^2^: 0.9730

Slope: -2.164

Cq of NTC: No amplification detected

Cq of no reverse transcriptase control: No amplification detected

Cq Mean: 19.44

Cq Standard deviation: 0.78

Dissociation curve:


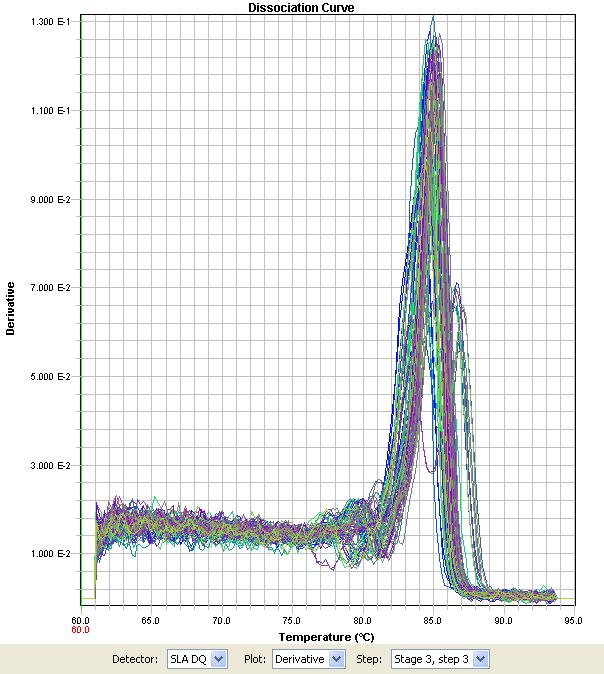


Sarcolipin

Gene symbol: SLN

Sequence ID: ENSSSCT00000030356

Forward primer: TGACGCTGTTCAGGAGATAAAGACC

Reverse primer: GGTGGATCGCTCCATTCTCAG

Amplicon length: 89 bp

Forward primer location: Spans boundary of exons 1 and 2

Reverse primer location: Within exon 2

Calibration curve:

Efficiency (from calibration curve): 1.00

R^2^: 0.9878

Slope: -2.328

Cq of NTC: No amplification detected

Cq of no reverse transcriptase control: No amplification detected

Cq Mean: 34.49

Cq Standard deviation: 1.75

Dissociation curve:


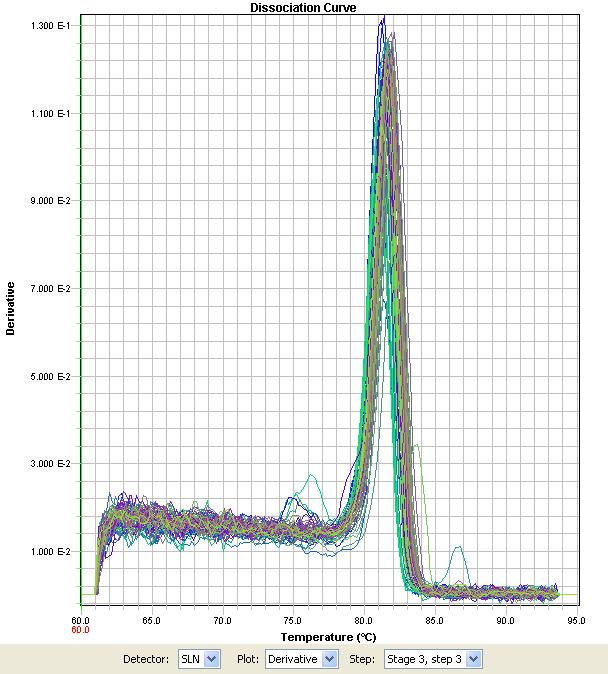


Transcobalamin 1

Gene symbol: TCN1

Sequence ID: ENSSSCT00000023981

Forward primer: AGCTATGCAGGCTCTCTTTGTCG

Reverse primer: TGGTAGACGGAATACTCCCTGAGATG

Amplicon length: 106 bp

Forward primer location: Spans boundary of exons 6 and 7

Reverse primer location: Spans boundary of exons 7 and 8

Calibration curve:

Efficiency (from calibration curve): 0.96

R^2^: 0.9956

Slope: -2.383

Cq of NTC: No amplification detected

Cq of no reverse transcriptase control: No amplification detected

Cq Mean: 32.81

Cq Standard deviation: 1.98

Dissociation curve:


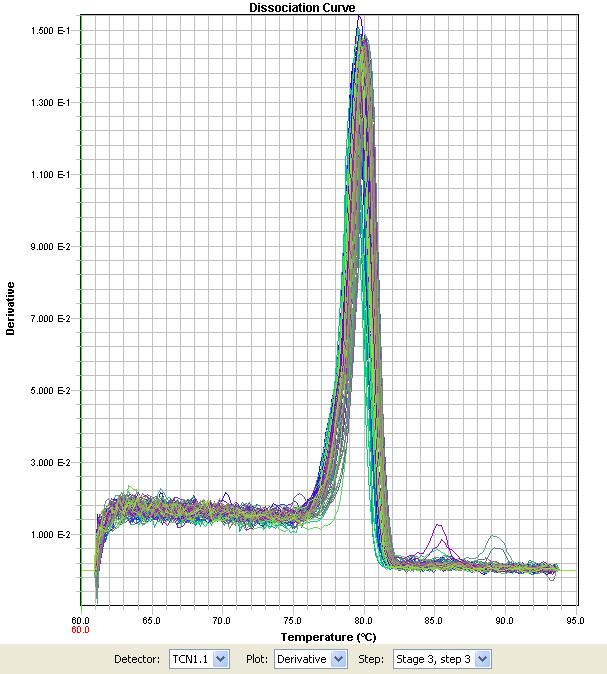


Troponin T type 1

Gene symbol: TNNT1

Sequence ID: ENSSSCT00000023274

Forward primer: ACCTGGTCAAGGCAGAACAGAAG

Reverse primer: GAGGCTTTTTACGCTCGGACAG

Amplicon length: 87 bp

Forward primer location: Spans boundary of exons 9 and 10

Reverse primer location: Within exon 10

Calibraton curve:

Efficiency (from calibration curve): 1.01

R^2^: 0.9982

Slope: -2.309

Cq of NTC: No amplification detected

Cq of no reverse transcriptase control: No amplification detected

Cq Mean: 34.18

Cq Standard deviation: 1.10

Dissociation curve:


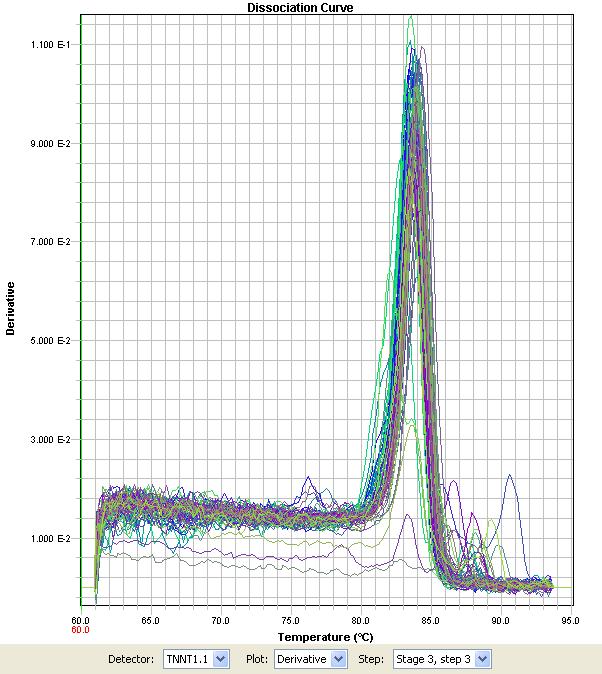

Supplement: S1 Appendix — (DOCX) [file pone.0138653.s001.docx]
